# Process Operability Analysis of Membrane-Based Direct Air Capture for Low-Purity CO_2_ Production

**DOI:** 10.1021/acsengineeringau.3c00069

**Published:** 2024-03-28

**Authors:** Vitor Gama, Beatriz Dantas, Oishi Sanyal, Fernando V. Lima

**Affiliations:** Department of Chemical and Biomedical Engineering, West Virginia University, Morgantown, West Virginia 26506, United States

**Keywords:** process design, membrane DAC, process operability, hollow fibers, CO_2_ capture

## Abstract

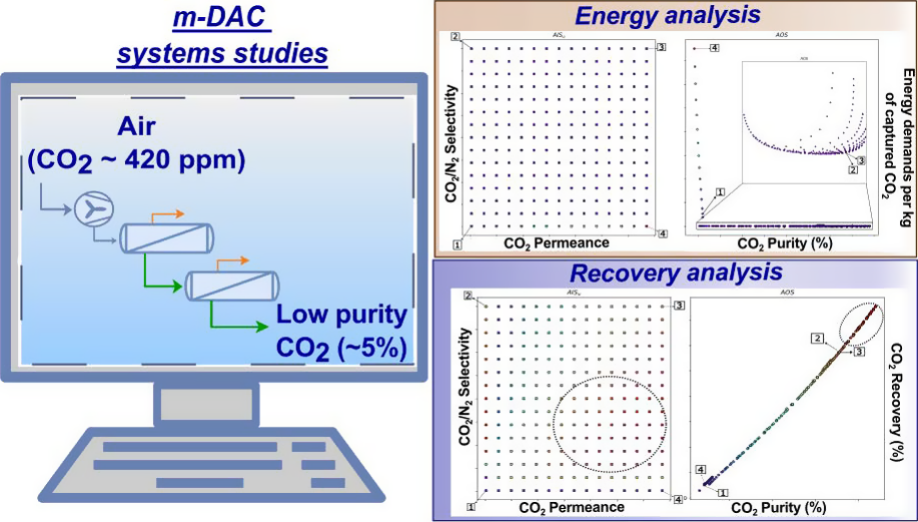

Addressing climate change constitutes one of the major scientific challenges of this century, and it is widely acknowledged that anthropogenic CO_2_ emissions largely contribute to this issue. To achieve the “net-zero” target and keep the rise in global average temperature below 1.5 °C, negative emission technologies must be developed and deployed at a large scale. This study investigates the feasibility of using membranes as direct air capture (DAC) technology to extract CO_2_ from atmospheric air to produce low-purity CO_2_. In this work, a two-stage hollow fiber membrane module process is designed and modeled using the AVEVA Process Simulation platform to produce a low-purity (≈5%) CO_2_ permeate stream. Such low-purity CO_2_ streams could have several possible applications such as algae growth, catalytic oxidation, and enhanced oil recovery. An operability analysis is performed by mapping a feasible range of input parameters, which include membrane surface area and membrane performance metrics, to an output set, which consists of CO_2_ purity, recovery, and net energy consumption. The base case for this simulation study is generated considering a facilitated transport membrane with high CO_2_/N_2_ separation performance (CO_2_ permeance = 2100 GPU and CO_2_/N_2_ selectivity = 1100), when tested under DAC conditions. With a constant membrane area, both membranes’ intrinsic performances are found to have a considerable impact on the purity, recovery, and energy consumption. The area of the first module plays a dominant role in determining the recovery, purity, and energy demands, and in fact, increasing the area of the second membrane has a negative impact on the overall energy consumption, without improving the overall purities. The CO_2_ capture capacity of DAC units is important for implementation and scale-up. In this context, the performed analysis showed that the m-DAC process could be appropriate as a small-capacity system (0.1–1 Mt/year of air), with reasonable recoveries and overall purity. Finally, a preliminary CO_2_ emissions analysis is carried out for the membrane-based DAC process, which leads to the conclusion that the overall energy grid must be powered by renewable sources for the technology to qualify within the negative emissions category.

## Introduction

Recent studies reveal that fossil fuel induced CO_2_ emissions contribute significantly to the rise in the global average temperature.^1^ Combating this would require not only reducing or mitigating emissions, but also addressing legacy emissions.

**Figure 1:**
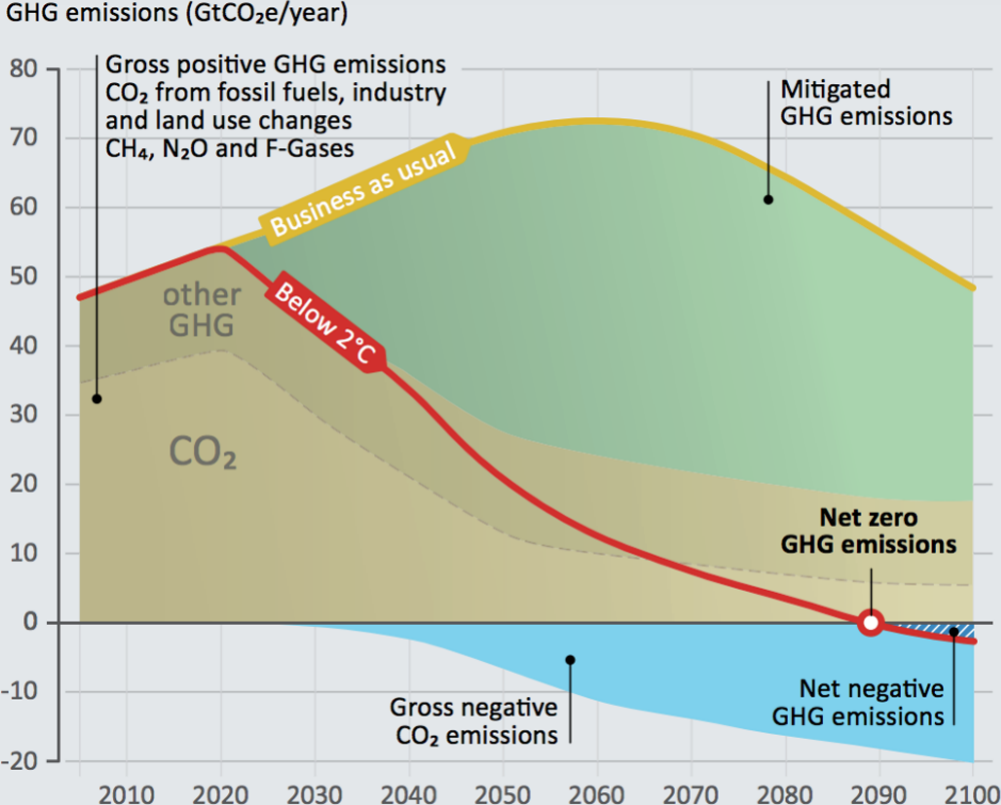
Impact of negative emissions technologies on reaching net-zero emissions.^2^

As seen in Figure 1, one of the envisioned ways to reach net zero is to develop negative emissions technologies such as CO direct air capture (DAC). Separation processes such as adsorption and absorption have been extensively developed for point-source capture (e.g., power plants) and are also the front runners in case of DAC. In fact, a number of commercial DAC plants employ these technologies, examples being amine-functionalized sorbents used by Climeworks and the KOH-based absorption process used by Carbon Engineering.^2,3^

Adding to the portfolio of separation technologies that can be applied for DAC, the use of gas separation membranes has also been considered in this context.^4−11^ Membrane-based direct air capture (m-DAC) offers several advantages such as simple setup and operations due to its modular design, allowing for easy adaptation to different capture unit sizes and geographical locations. Additionally, membranes eliminate the need for a regeneration step, potentially reducing the energy costs associated with it. For these reasons, the US Department of Energy (DOE-NETL) has also invested in evaluating the m-DAC process using a hybrid polymer flat-sheet membrane (HypoMem).^9^ The biggest challenge in implementing membranes for DAC is the low CO_2_ partial pressure in the air, which translates into a low driving force for the permeation process. Compression of the feed stream to increase the driving force is not energetically favorable; therefore, using downstream vacuum is required to provide the necessary driving force.^12^ The process naturally becomes pressure-ratio-limited, and thus recovering high-purity (>90%) CO_2_, as is possible with sorption processes, would require multiple stages. Despite the many advantages of membrane-based DAC processes as listed above, this is a clear disadvantage of this route and it is unlikely that it could be competitive with the sorption processes at the kiloton/megaton scales. In this paper, the authors investigate a specific scenario to enable m-DAC, wherein a membrane-based process is used to produce a low-purity (≈5%) CO_2_ stream.

Low-purity CO_2_ could be applied specifically as a yield boost in algae, urea, and fertilizer production, among other applications.^13^ Techno-economic and life cycle analyses demonstrated the capabilities of redirecting CO_2_ from flue gas generated in a coal-fired power plant as algae feedstock. CO_2_ concentration in the waste flue gas ranges from 5 to 15%, and the 30-year life-cycle analysis reported the net capture of 1.16 × 10^4^ metric tons of CO_2_.^14^ It has been demonstrated that 5% CO_2_ is optimal for specific algae usage.^15^ Other applications of low-purity CO_2_ streams include enhanced oil recovery and oxidant stream for catalytic processes.^16^ This study specifically investigates the feasibility of an m-DAC process for low-purity CO_2_ production by using a process operability framework.

Experimental demonstrations of membranes for direct air capture (m-DAC) are limited. In this context, the facilitated transport membranes developed by Lee et al.^17^ are a notable example of DAC membranes. These membranes were created using poly(ionic liquid)-ionic liquid/graphene oxide (PIL-IL/GO) and when used with ambient air (≈410 ppm of CO_2_, balance N_2_, O_2,_ and humidity) as feed, showed CO_2_ permeance of 2100 GPU with CO_2_/N_2_ and CO_2_/O_2_ selectivities of 1100, and 265, respectively. However, for higher CO_2_ feed concentrations (≈10,000 ppm of CO_2_), it showed lower CO_2_ permeance of ≈128 GPU and CO_2_/N_2_ and CO_2_/O_2_ selectivities of 68 and 17, respectively. One GPU (Gas Permeation Unit) 

The decline in performance for facilitated transport membranes for higher CO_2_ partial pressure conditions has been reported in prior literature, and the separation mechanism is closer to solution diffusion under these conditions. In this paper, the feasibility of an m-DAC process design is evaluated for low-purity CO_2_ production using a hollow fiber membrane setup in AVEVA Process Simulation. Hollow fiber configurations provide high surface area-to-volume ratios and millions of such fibers can be easily packed into modules, thus enabling high-productivity separation devices. The membrane properties noted by Lee et al.^17^ are used in the base case scenario of this hollow fiber membrane simulation. The analysis of the proposed process is then performed by employing a process operability framework. Process operability is a tool for qualitatively and quantitatively assessing process design and control in the early stages of the process.^18−21^ It can evaluate process performance in terms of achieving desired operating conditions while constraining input and output variables within specific ranges. The results collected from the base case scenario are used in the operability analysis to identify the important trends in the systems-level performance, with respect to the membrane’s intrinsic properties and design specifications.

By addressing the gap in the application of membranes for DAC, as underscored elsewhere,^5,6^ this study provides a framework on membrane use for DAC through process operability analysis by relating membrane module properties to product purities and recoveries. Ultimately, this framework is expected to guide future experimental efforts in membrane development. Finally, an energy analysis of the m-DAC process is performed using publicly available information on the US energy grid considering both renewable and fossil-based sources.^22^ Thus, this work provides a platform that can be employed for further systems studies toward enabling m-DAC technologies for decarbonization.

## Background

### Membrane Process Description

The performance of membrane materials is primarily described by two parameters: permeability (or permeance) and selectivity. The permeability, which quantitatively describes the relative ease with which a gas molecule passes through a membrane, is expressed in terms of the flux and the pressure driving force, as noted in eq 1.
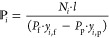
1In which, *N*_*i*_ represents the molar flux of component *i*, and *P*_f_ and *P*_p_ are the pressures in the feed and permeate sides in [bar], respectively. *y*_*i*,f_ is the fraction of component *i* in the feed, while *y*_*i*,p_ is the component’s fraction on the permeate side. *l* represents the membrane selective layer thickness in [mm], and, , is the permeability of the component in the membrane material in [kmol/m^2^·h·bar]. The permeability property is modeled differently depending on the type of membrane. Solution-diffusion membranes, for instance, consider that the permeant molecules will dissolve in the material and then diffuse based on a concentration gradient.^23^ However, their performance is limited by a trade-off relationship between permeability and selectivity, as highlighted by Robeson.^24^ In contrast, facilitated transport membranes can overcome this barrier due to the specialized carriers present in the membrane layer. These carriers can interact with the component of interest thus enhancing its permeation rate.^25,26^

The membrane’s selectivity is a measure of how certain components will preferentially permeate through the material when compared to other components present in the mixture. Considering *i* as the faster diffusing component and *j* as the slower one, the expression for selectivity is shown in eq 2.
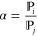
2In addition to the membrane’s intrinsic properties, three important performance parameters should be highlighted: stage cut, component recovery, and permeate purity. The latter refers to the CO_2_ mole fraction in the permeate, *y*_*i*,p_, while stage cut, θ, refers to the ratio of the total permeate flux, *N*_p_, to the feed flux, *N*_f_.
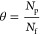
3For any individual component *i*, its recovery, *R*_*i*_, is defined as
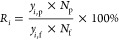
4

### Operability Framework

In process operability, a process model or simulation, *M*, describes the relationship between the inputs (physical sizes and manipulated variables) and the outputs:
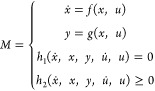
5

In which, in the given system *M*, m inputs are represented by *u* ∈ , *p* outputs by *y* ∈ , and *n* states variables by *x* ∈ . *f* and *g* are nonlinear maps, and *h*_1_ and *h*_2_ correspond to equality and inequality process constraints, respectively. The manipulated inputs, *u*, assume that specific ranges in the available input set (AIS) are being conditioned by design or operational constraints. The AIS is given by
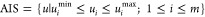
6

For example, in this work, CO_2_ permeance, CO_2_/N_2_ selectivity, and membrane surface areas are considered in this input set. Based on the process model, the achievable output set (AOS) is defined by a set of reachable outputs, *y*, that can be achieved using the inputs inside the AIS as follows:
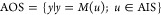
7In this paper, CO_2_ purity and recovery, as well as energy demands, are elements of this output set.

## Methods

### m-DAC Process Simulation Setup

This study uses a first-principles equation-oriented model for a hollow-fiber membrane module available in AVEVA Process Simulation to analyze the application of membrane separation for DAC.^27^ The model discretizes the membrane in the length direction and solves the mass and energy balances for each incremental element, considering a shell and tube-like geometry, similar to the method implemented by Bishop et al.^27^

Following the approach proposed by Fujikawa et al.,^6^ where a multistage membrane process was studied for DAC, the proposed m-DAC process design is set as two membrane modules arranged in series, with the air feed at atmospheric conditions and the permeate is maintained under vacuum conditions.

**Figure 2:**
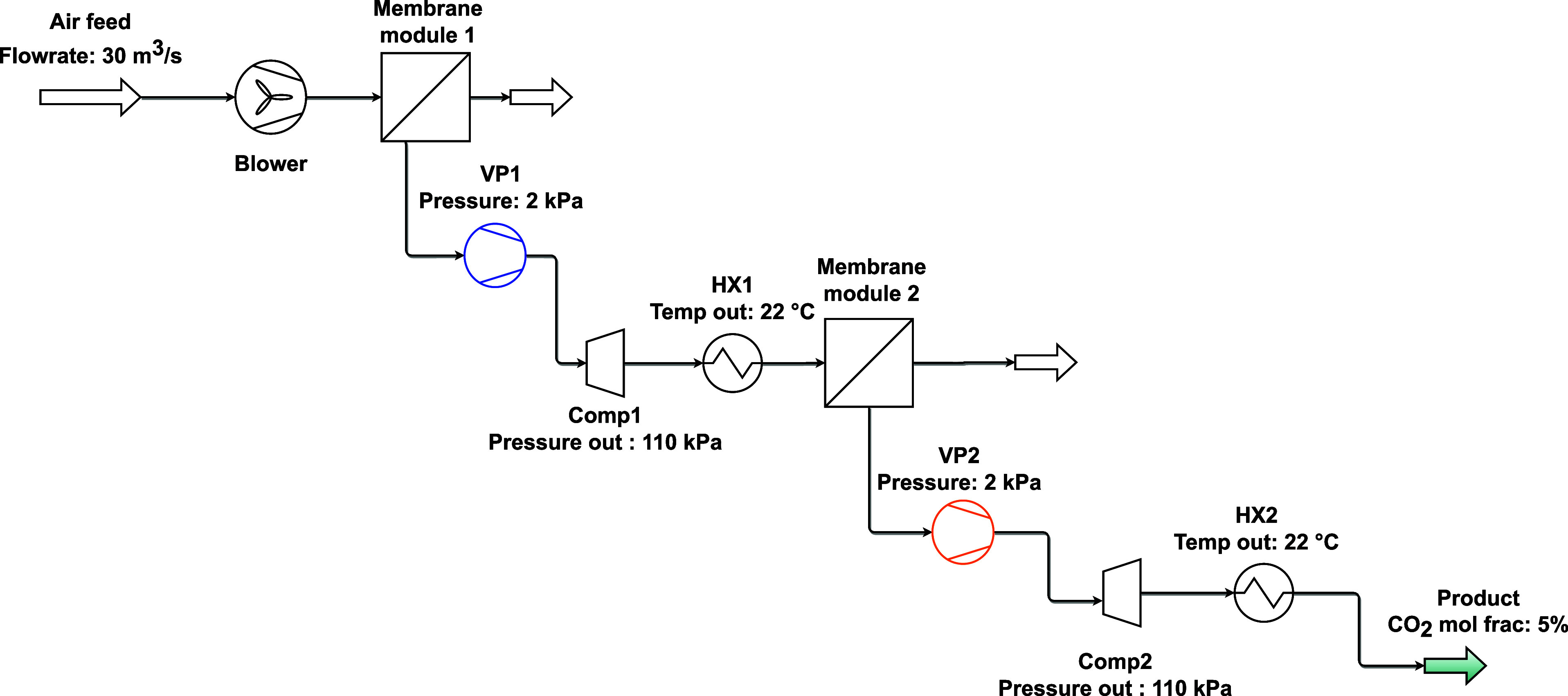
m-DAC proposed flowsheet.

The capture goal in this study is a permeate product with ≈5% CO_2_ since this purity level has been shown to be optimal for algae applications.^15^ The membrane properties and process conditions employed are described in Tables 1 and 2, respectively, while the process flowsheet is exhibited in Figure 2. The base case feed flow rate was set to 30 m^3^/s (i.e., 1 Mega-ton of air/year).

**Table 1:** m-DAC Feed Properties (Humidity Not Considered)

parameter	feed
air flow rate [m^3^/s]	30
temperature [°C]	22
pressure [kPa]	110
mole frac [%]	
nitrogen	0.79
oxygen	0.21
carbon dioxide	0.000420

**Table 2:** Membrane Module Characteristics

parameter	membrane module 1	membrane module 2
fiber ID [μm]	100	100
fiber OD [μm]	200	200
fiber length [m]	5	5
shell ID [m]	5	5
selective layer thickness [μm]	1	1
Permeance [GPU]		
CO_2_	2100	128
N_2_	≈2	≈2
O_2_	≈8	≈8
Selectivity		
CO_2_/O_2_	265	≈17
CO_2_/N_2_	1100	68

For simplicity, hollow fiber geometries were considered for the membrane modules for both stages. However, one could consider different geometries for different stages, but such optimizations are beyond the scope of this paper. The membrane intrinsic properties (e.g., permeance and selectivity) in Table 2 are assumed to be the same as reported by Lee et al.^17^ It must be noted that this facilitated transport membrane was developed as a flat sheet and has not been reported to be configured into hollow fibers. Therefore, one of the key assumptions in this study is that a thin selective layer of PIL-IL/GO can be coated on a porous support, as reported elsewhere,^28^ and the resulting high-performing material can be formatted into hollow fibers. The selective layer, in this case, is assumed to be ≈1 μm (1 μm = 10^–6^ m), which is a reasonable assumption, even though thinner (≈ 0.5 μm) selective layers have been reported elsewhere.^29^

The “Opyrability” package,^30^ developed in Python, was applied to investigate the direct mapping of the inputs, CO_2_ permeance, and CO_2_/N_2_ selectivity to the outputs (energy demands, purity of the product, and its recovery) by the discretization of the AIS, using their values in the steady-state model to generate the AOS.

## Results and Discussions

This work describes a hollow-fiber membrane-based process design that extracts CO_2_ from ambient air and concentrates it to an ≈5% permeate CO_2_ level. The base case of this simulation work assumes the hollow fiber properties to be equivalent to those of the PIL-IL/GO-facilitated transport membrane described by Lee et al.,^17^ i.e., CO_2_ permeance nominal value of 2100 GPU (7.0350 × 10^–7^ kmol·m^–2^·s^–1^·kPa^–1^) and CO_2_/N_2_ and CO_2_/O_2_ selectivities of 1100 and 265, respectively, for the first membrane module and CO_2_ permeance of 128 GPU (4.288 × 10^–8^ kmol·m^–2^·s^–1^·kPa^–1^) and CO_2_/O_2_ and CO_2_/N_2_ selectivities of 17 and 68, respectively, for the second module. Using the information from the base case, a process operability analysis is performed to assess the impact of the effects of input parameters (membrane properties, module surface areas, and feed flow rate) on the final CO_2_ purity, CO_2_ recovery, and energy consumption. This analysis helps to identify the critical parameters that impact the system’s performance and areas of improvement in terms of membrane material and process design.

### Base Case

Leveraging AVEVA’s Process Simulation equation-oriented solver, basic membrane features (e.g., number of fibers, stage cut, etc.) were determined such that the simulation would achieve 5% CO_2_ in the final product. In order to achieve the Megaton scale of processed air, a 30 m^3^/s feed flow rate (i.e., 0.012 m^3^/s of CO_2_ = 420 tons/year of CO_2_) is set for the process, and, two stages were deemed necessary in this case to reach 5% CO_2_ in the product. Targeting a low-purity CO_2_ product minimizes the number of stages required, when compared against the four-stage system discussed by Fujikawa et al.^6^ This base case resulted in an overall CO_2_ recovery of 1.3% (6.62 tons/year), with the final product composition being ≈5% CO_2_, ≈24% N_2,_ and 70% O_2_. The required membrane areas and number of fibers needed are calculated based on the design conditions, highlighted in Table 2, and the purity goal. The computed values are exhibited in Table 3, and the operable regions of the proposed process are analyzed in the following section, discussing the effects of operations and intrinsic parameters on CO_2_ capture.

**Table 3:** Membrane Module Calculated Parameters

parameter	calculated value
number of fibers (1st membrane module)	1,000,000
number of fibers (2nd membrane module)	283,985
first membrane module surface area [m^2^]	3171.4
second membrane module surface area [m^2^]	900.6

### Case Studies Description

Three specific case studies are discussed in this paper: impacts of (a) membrane intrinsic properties, (b) membrane surface areas, and (c) feed flow rate. For case studies 2 and 3, described in Table 5, the AIS design space bounds were selected as ±30% of the base values, while for Case 1, the bounds are based on reported values in the literature. Table 4 details the AIS values for each of the case studies performed.

**Table 4:** Variables Selected for the Available Input Set (AIS)

variable	base case value	lower bound	upper bound
membrane module 1 area [m^2^]	3171.4	2220	4122.8
membrane module 2 area [m^2^]	900.6	630.4	1170.8
CO_2_ permeance (1st module) [GPU]	2100	100	20,000
CO_2_ permeance (2nd module) [GPU]	128	100	2000
CO_2_/N_2_ selectivity (1st module)	1100	8	2000
CO_2_/N_2_ selectivity (2nd module)	68	8	200

The abovementioned AIS variables are mapped through the process model, and the results collected for the AOS are shown in Table 5.

**Table 5:** Operability Case Studies

case studies	AIS	AOS
1	CO_2_ permeance and CO_2_/N_2_ selectivity	CO_2_ purity vs energy requirements, CO_2_ purity vs CO_2_ recovery
2	membrane module surface areas	CO_2_ purity vs energy requirements, CO_2_ purity vs CO_2_ recovery
3	feed flow rate	CO_2_ purity vs CO_2_ recovery

### Operability Studies

#### Case 1 – Impact of Changes in Membrane Properties on System Performance

In this section, the membrane’s intrinsic characteristics effects on the AOS are investigated. The AIS bounds for the first case study are defined to cover a broad range of membrane performances, as highlighted in Table 2. This design space was formulated so that it does not significantly exceed the realistic range of permeances reported in the literature. The upper bound of CO_2_ permeance (20,000 GPU) is based on the value suggested by Fujikawa et al.^6^ While such performance has only been reported for nanomembranes,^31^ it is possible that some of the intrinsically high-performance membrane materials reported in the literature, when configured into hollow fibers with ultrathin (≈100 nm) selective layers, could yield such high permeance.^32^

The lower bound of 100 GPU has been reported for a blend membrane composed of carboxymethyl chitosan (CMC) and poly(amidoamine) (PAMAM) in the literature.^33^ The lower bound of CO_2_/N_2_ selectivity approximately was chosen to be close to the Knudsen selectivity value (≈1.8). The upper bound for selectivity exceeds literature values; however, as facilitated transport membranes such as the PIL-IL/GO membranes continue to be developed, attaining such high selectivities in the near future is not unreasonable to expect. In all cases, the CO_2_/O_2_ selectivity was maintained to be 0.25 × CO_2_/N_2_ selectivity, to closely model the behavior of the reported facilitated transport membrane by Lee et al.^17^ In future work, the variation of the CO_2_/O_2_ selectivity with the CO_2_ permeance must be considered to match more realistic scenarios.

**Figure 3:**
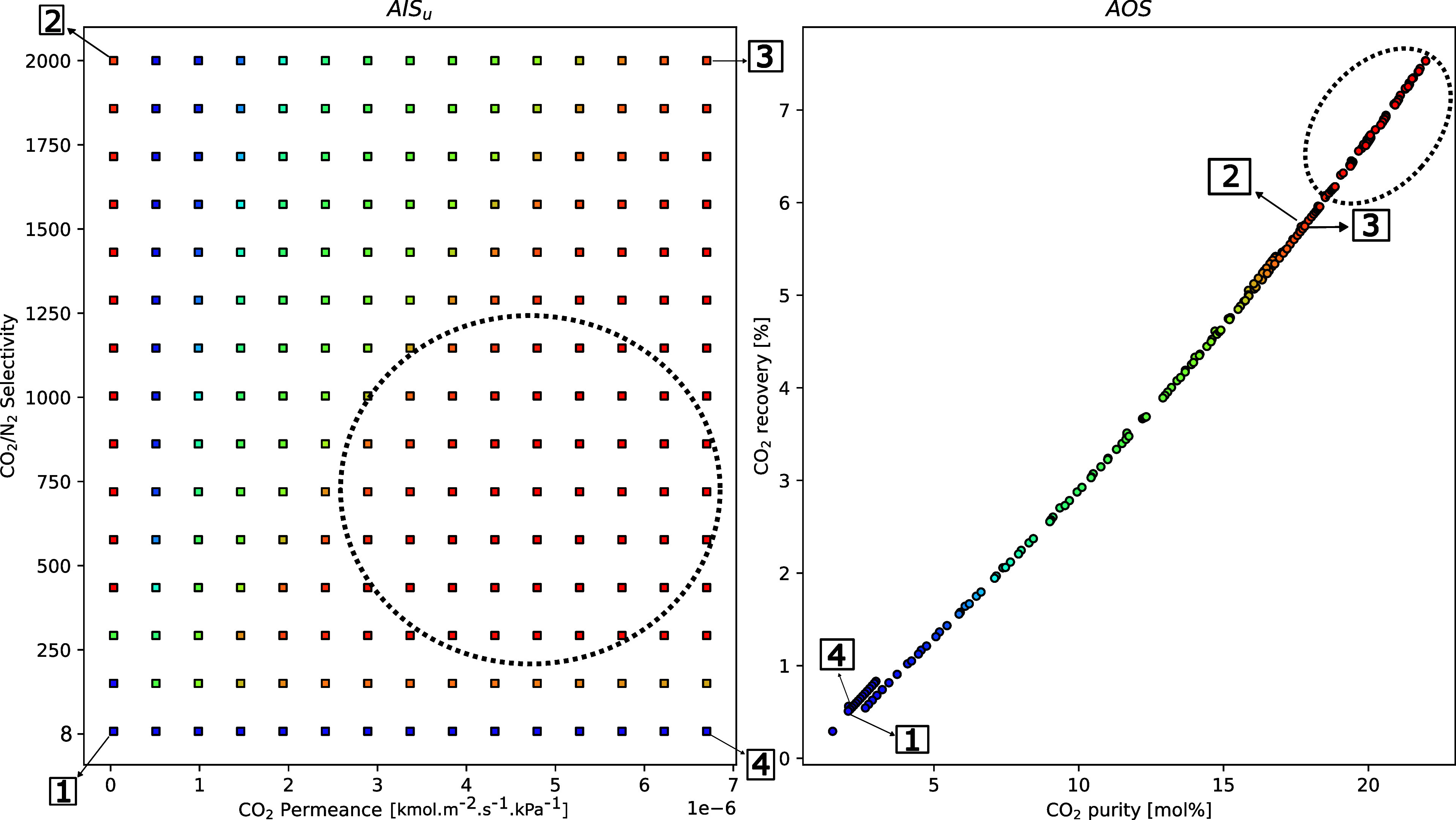
Case 1.1**—** m-DAC operability study investigating the first membrane module permeance and selectivity on the overall CO_2_ recovery and purity. Points 1, 2, 3, and 4 denote the four bounds of the AIS - [1] low CO_2_ P/L; low CO_2_/N_2_ selectivity, [2] low CO_2_ P/L; high CO_2_/N_2_ selectivity, [3] high CO_2_ P/L; high CO_2_/N_2_ selectivity, and [4] high CO_2_ P/L; low CO_2_/N_2_ selectivity. (1 GPU = 3.35 × 10^–10^ kmol m^–2^ s^–1^ kPa^–1^).

**Figure 4:**
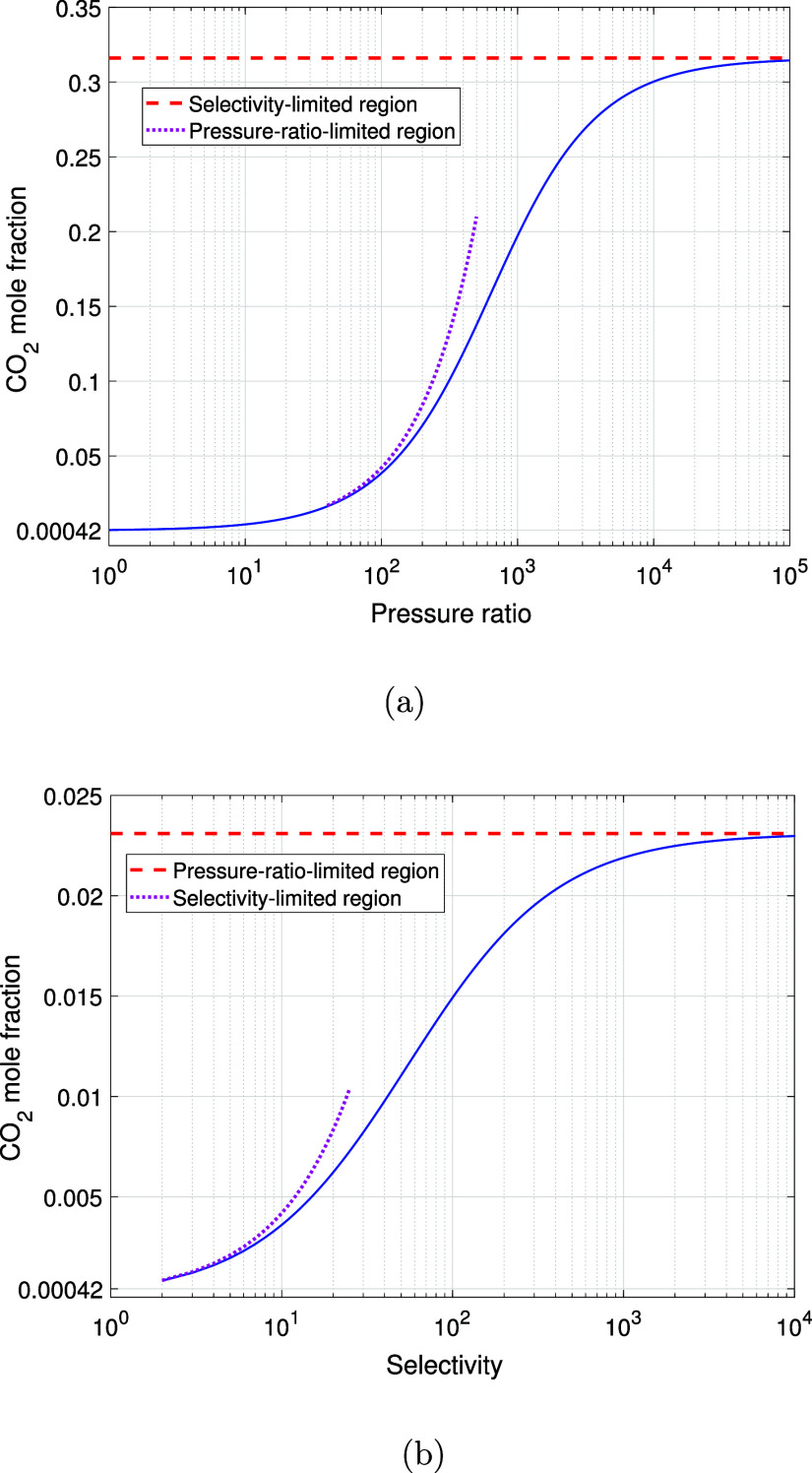
Theoretical CO_2_ mole fraction analysis. (a) Pressure ratio and CO_2_ mole fraction relationship at fixed selectivity (1100); (b) selectivity and CO_2_ mole fraction relationship at fixed pressure ratio (55).

As shown in Figure 3, the AOS results indicate that improvements in the CO_2_ permeance and CO_2_/N_2_ selectivity of the first membrane lead to predictable improvements in the capture. Nevertheless, by analyzing points 2 and 3 in the AOS, it is clear that for the highest selectivity values considered here, the effect of permeance appears to be negligible. A similar conclusion can be drawn from the analysis of the low-selectivity (1, 4) values; however, these values correspond to low CO_2_ purity and recovery. It is interesting to note that the best recovery purity results are provided by membranes with properties representative of the circled area in Figure 3. This implies a nonlinear relationship between the final product and the properties of the first membrane module. It should be noted that the final purity at the end of the first stage reaches only ≈2% (Figure S1) despite using highly selective membranes. This can be explained by the fact that this process is inherently pressure-ratio-limited, as discussed in the context of Figure 4. In the Supporting Information, we have provided the analysis of the individual membrane properties’ effects on the permeate stream of each stage (Figures S1 and S3). Comparing these results with those in the context of Figure 3, it is possible to see that these trends are unlike each other, which highlights the complexity of coupling multiple membranes with different properties.

The pressure ratio (φ = *P*_f_/*P*_p_) of the proposed process is consistently set at 55 for both membrane modules, and this plays a key role in determining the final purity, as shown in Figure 4.^23^Figure 4 displays the relationship between the pressure ratio designed for the proposed process, as well as the selectivity relationship with the permeate CO_2_ mole fraction. It is important to note that improvements in CO_2_ concentration are small when approaching the pressure-ratio-limited region. However, working in this region was of interest in this research since increasing the pressure ratio would require increasing the upstream pressure, which in turn would involve compressing the feed (air) or further decreasing the vacuum pressure on the downstream. Neither of these options are economical, thus we are restrained to the pressure-ratio-limited region. The pressure ratio, as discussed by Huang et al.,^34^ impacts the permeate composition directly since it limits the maximum reachable concentration of the desired component. For the membrane separation designed in this study, the pink dotted line in Figure 4 and the red dashed line in Figure 4 mark the region where the effects of the pressure ratio can limit the theoretical concentration achievable by a single membrane module when considering the membrane used in this work. This explains why, despite using high permeance membranes, the purity reaches ≈2% at the end of the first stage.

**Figure 5:**
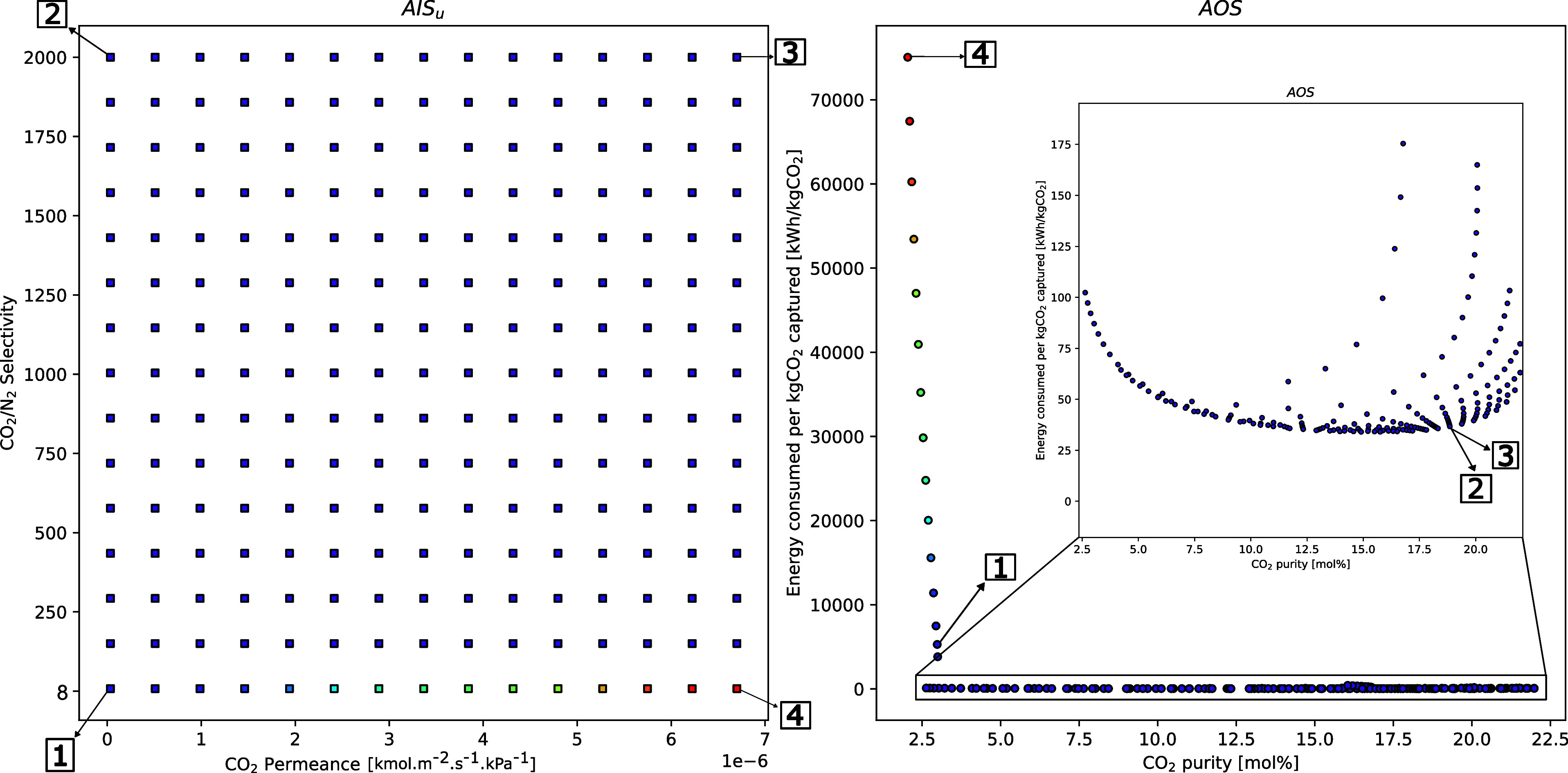
Case 1.2**—** m-DAC operability study investigating the permeance and selectivity influence on the CO_2_ recovery and energy consumption. Points 1, 2, 3, and 4 denote the four bounds of the AIS - [1] low CO_2_ P/L; low CO_2_/N_2_ selectivity, [2] low CO_2_ P/L; high CO_2_/N_2_ selectivity, [3] high CO_2_ P/L; high CO_2_/N_2_ selectivity, and [4] high CO_2_ P/L; low CO_2_/N_2_ selectivity. (1 GPU = 3.35 × 10^–10^ kmol m^–2^ s^–1^ kPa^–1^).

Additional research was carried out to examine the influence of the intrinsic properties of the second membrane module on the final CO_2_ recovery and purity, and these results are provided in Figure S3. The findings indicated that the properties of the second membrane had minimal impact on the desired product recovery, establishing the first module as the most dominant step in determining the recovery. The purity, however, could be significantly enhanced by the selectivity of the second membrane module. Practically however, it could be challenging to achieve such high CO_2_/N_2_ selectivities (≈200) at high feed concentrations in facilitated transport membranes. In terms of energy consumption, it is observed that as CO_2_/N_2_ selectivity increases, energy consumption drastically reduces. At high selectivities, purity is maximized when permeance is also increased as demonstrated by the point [3] in the above figure. This outcome aligns with the literature, where selectivity has been shown to exert a strong influence on the energy demands of an m-DAC process.^6^ These observations related to Figures 3 and 5 collectively underscore the intricate interplay between selectivity, energy consumption, and purity for the m-DAC system. Similar trends were observed for the isolated membrane cases, where the CO_2_/N_2_ selectivity for each membrane impacted the energy requirements for that stage, as shown in Figures S2 and S4.

#### Case 2—Impact of Changes in the Membrane Surface Area on System Performance

In the next case study, a 2 × 3 (inputs x outputs) subsystem operability analysis was conducted, with the inputs, in this case, being the membrane surface areas of the two modules. The membrane performance values were fixed at the base case simulation values.

**Figure 6:**
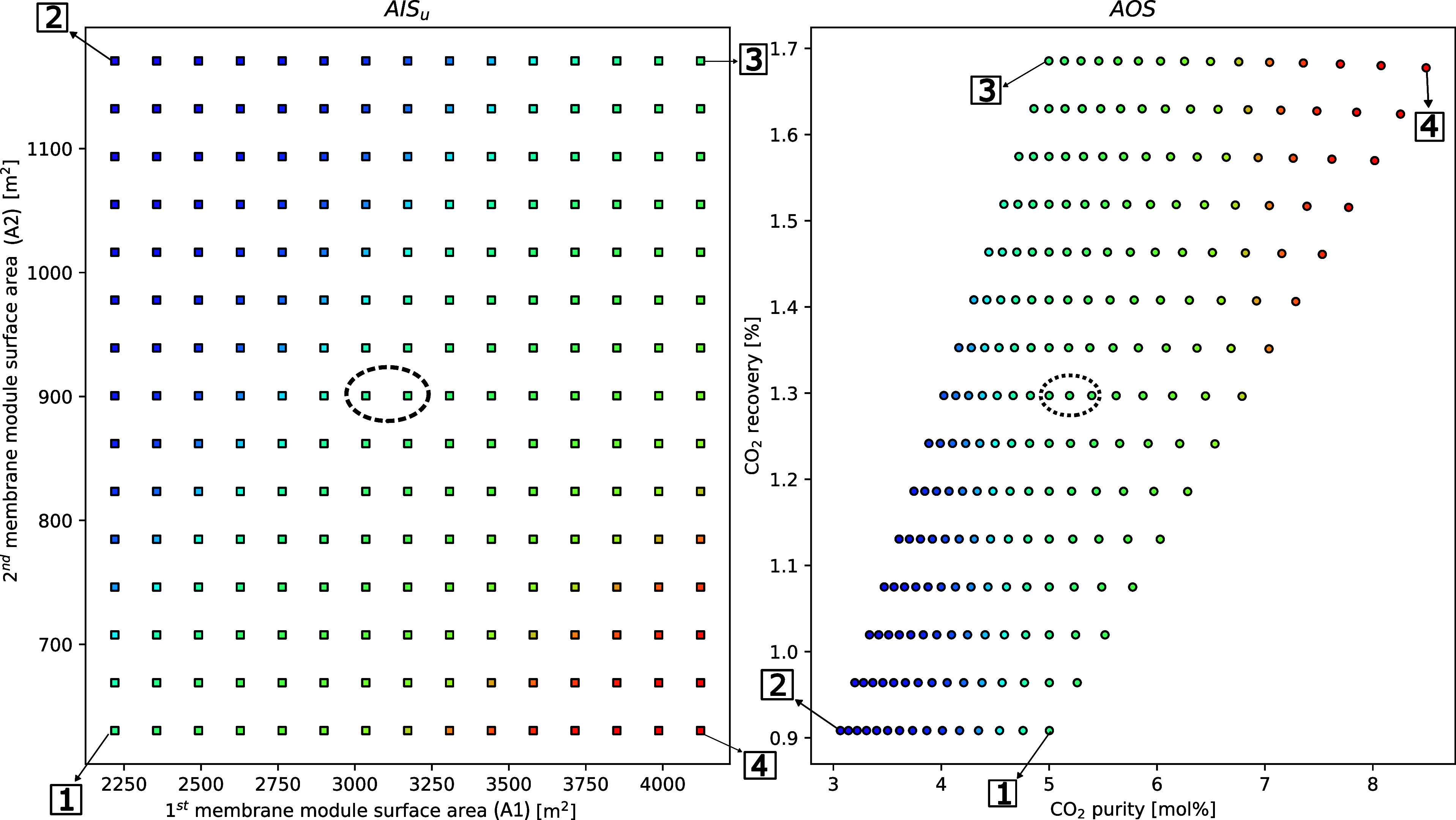
Case 2.1—m-DAC operability study investigating the influence of membrane surface areas on CO_2_ recovery and purity. The points 1, 2, 3, and 4 denote the four bounds of the AIS - [1] low A1 and A2; [2] low A1 and high A2; [3] high A1 and A2; and [4] low A2 and high A1. The circles highlight the base case conditions and results.

**Figure 7:**
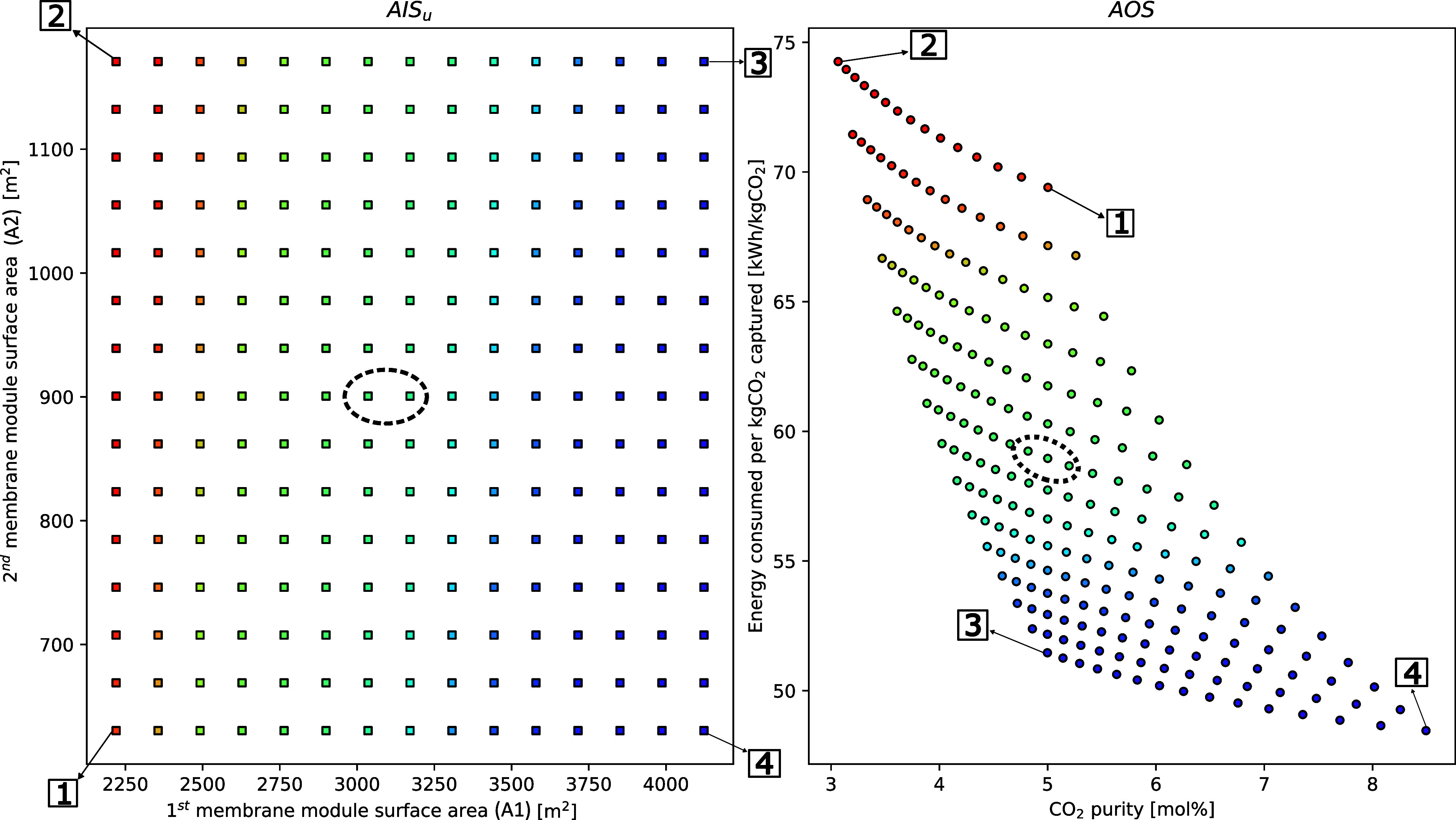
Case 2.2—m-DAC operability study investigating the influence of membrane surface areas on CO_2_ recovery and energy consumption. The points 1, 2, 3, and 4 denote the four bounds of the AIS—[1] low A1 and A2; [2] low A1 and high A2; [3] high A1 and A2; and [4] high A1 and low A2. The circled areas highlight the base case conditions and results.

The recovery values achieved in this case study range from ±30% of the base case recovery (1.3%), closely following the changes in surface area. On the other hand, as seen in Figure 6, the CO_2_ purity exhibited a linear trend, achieving purities that fall above and below the ±30% of base case CO_2_ achieved. On assessing the impact of each module in the capture, it is clear that the first module has a more significant impact than the second module on attaining high purities and recoveries. This is fairly intuitive, since the first module deals with a very high volume of air with only 420 ppm of CO_2_, thus acting as a concentrator. Conversely, increasing the surface area of the second module does not yield significant improvements and can, in fact, increase the energy demands of the process, as evident by points [2] and [3] in Figure 7.

For the base case, the energy consumed and purity are 57 kWh/kgCO_2_ and 5%, respectively, as shown in Figure 7. Findings for this case reveal that operating with the largest available surface area for both modules, point [3], results solely in increased energy consumption with no discernible effect on purity. To achieve enhanced purities, as previously discussed, it is imperative to maintain a larger surface area for the first membrane module while reducing the surface area of the second module, as is the case of point [4]. This arrangement positively impacts purity while simultaneously reducing energy consumption. In the case of hollow fiber membrane modules, increments in the available surface area could be achieved by increasing the module length or by increasing the packing density of fibers within the modules. In fact, one of the advantages of hollow fiber configuration over flat sheets or spiral wound membranes is their high surface area to volume ratio (as high as 10,000 m^2^/m^3^). This facilitates highly effective surface areas while still retaining the compact and modular nature of the membrane systems.

#### Case 3—Impact of Feed Flow Rates on the System Performance

In case studies 1 and 2, the target air feed flow rate was maintained at ≈1 Mt/year (≈43 tons CO_2_/year) capacity, and for these cases, the maximum CO_2_ recovery was ≈8% and the purity 23%. As discussed in the context of the area analysis in the earlier section, the first module has to process large volumes of air, which could compromise the overall recovery and purity. In this section, the effect of the overall feed volume processed by the two-stage system on the product quality and recovery was assessed to understand if the m-DAC system could perhaps be more suited for smaller capture systems with lower than Mt capacity. The feed values considered for this investigation are 300 m^3^/s (10.2 Mt air/year, 430 tons CO_2_/year), 3 m^3^/s (0.102 Mt air/year, 4.3 tons CO_2_/year), and 0.3 m^3^/s (0.01 Mt air/year, 0.43 tons CO_2_/year) of atmospheric air. For this particular case study, the membrane properties were assumed to be that of the base case, and the areas for the two modules were manipulated between ±30%, similar to the AIS from the section.

**Figure 8:**
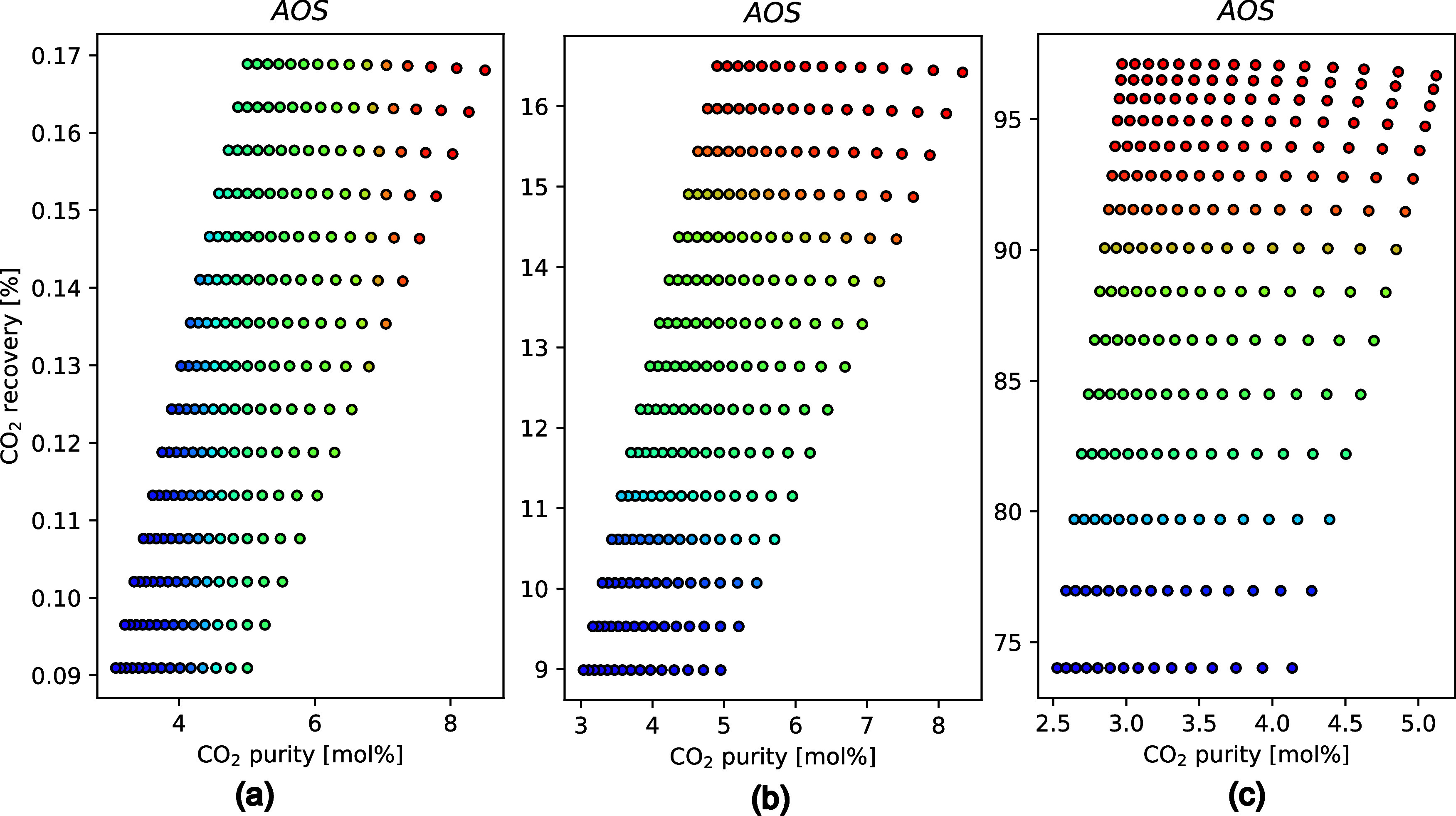
Feed loading influence on the CO_2_ purity and recovery. (a) Feed = 300 m^3^/s, (b) Feed = 3 m^3^/s, and (c) Feed = 0.3 m^3^/s.

The recovery trends with respect to the feed flow rates are as expected, where high recoveries (≈100%) are achieved under low feed flow rates. The purities are, however, limited to ≈8% even when the feed flow rate was set to 0.3 m^3^/s, as exhibited by Figure 8. The AIS variation presumably needs to be manipulated to achieve higher purities in case (c) by compromising the recoveries. In any case, the capacity for case (c) is too low and is not attractive for capture applications. The m-DAC process, thus, appears to be better suited for processing (0.1–1) Megaton/year of air (4–40 tons CO_2_/year) using the scheme proposed here, to achieve at least 8% or more recovery. Recovery systems with larger capacity than this must be either split into smaller parallel units or used for handling smaller volumes. Additionally, the optimum configuration for each of the modules could be identified in future studies, in which in this optimal structure the second membrane module could exhibit a more significant effect.

#### Negative Emissions Analysis

In this section, the prospect of the m-DAC process being a “negative emissions” approach is evaluated by considering the average energy requirements for the base-case m-DAC process and the CO_2_ emissions associated with various energy sources. The emissions are assessed using the ratio between the captured amount and the emitted CO_2_ resulting from energy production. Ratios > 1 indicate that the capture is not energetically efficient, and the net balance between captured and consumed is positive. To be considered carbon-neutral, this ratio should be 1, and for negative emissions, the value should be greater than 0 and less than 1. Three electricity sources the US grid, solar, wind, and their respective carbon intensity factors are considered for this analysis here, as highlighted in Table 6.

**Table 6:** Electricity Requirements and CO_2_ Emissions Based on Different Sources for the Proposed m-DAC

hourly capture [kgCO_2_ h^–1^]	16.53
capture capacity per year [tCO_2_ year^–1^]	6.62
electricity requirements [kWh]	57.73
electricity requirements per year [kWh year^–1^]	4.09 × 10^5^
CO_2_ Emissions and Capture Ratio^2^ (Emitted CO_2_/Captured CO_2_)	
US grid (390gCO_2_/kWh)^35^	1.36
solar (25gCO_2_/kWh)^2^	0.09
wind (11gCO_2_/kWh)^2^	0.04

As expected, when using the electricity provided by the US grid, which is generated primarily by natural gas (40%) and coal (19.5%),^22^ the proposed process achieves neither the net-zero nor negative-emissions scenario.^2^ Even though 20% of the energy in the US grid comes from renewable sources, the proposed m-DAC would still emit approximately 153 tons of CO_2_ per year.^22^ In contrast, the emission levels dropped significantly when the m-DAC process was powered by solar/wind sources. This drop indicates that if a larger portion of the current US grid would come from renewables, then it could be possible to operate and expand DAC technologies as negative emissions processes.

#### Perspective on the m-DAC Process for Low CO_2_ Production

Membrane Technology Research (MTR) recently demonstrated a membrane system for point source capture, and it is expected to capture ≈150 tonnes/day. The capture capacity is clearly significantly higher than the m-DAC system (6.62 tons of CO_2_/year). However, flue gas with 10% CO_2_ in it is ∼300 times more concentrated than ambient air, which translates to a higher driving force. Thus, even with a high-performance facilitated transport membrane described here, the recoveries are modest in the case of DAC versus point-source capture. A comparison of the m-DAC process with absorption or adsorption processes for low-purity CO_2_ production would be necessary to demonstrate if the former poses specific advantages in terms of lowering energy consumption. Facilitated transport membranes, such as those considered here, are perceived to be one of the most promising classes of membrane materials reported in the literature. Figure 3, however, demonstrates that membrane materials with even higher performance (>20,000 GPU) are needed to facilitate higher overall capture. Such material development should be coupled with superior hollow fiber fabrication strategies with defect-free ultrathin skin layers. The area requirements for each of the modules are very high; however, hollow fiber configurations are the most appropriate for such applications, in comparison to flat sheets or spiral wound membranes. As noted earlier, m-DAC is not suitable for megaton/gigaton CO_2_ capture rates, unless broken down into several parallel schemes. Furthermore, $100/ton of CO_2_ is largely accepted as the target operating ceiling for DAC operations. A techno-economic superstructure optimization could shed light on whether m-DAC systems could remain within this target. One of the applications noted in this work for low-purity CO_2_ streams is its use in algae growth; the high proportion of the O_2_ (70%) in the final stream could, however, pose challenges to its direct usage. Expanding the scope of the application of low-purity CO_2_ streams could thus motivate the development of m-DAC processes. Furthermore, the hybridization of m-DAC with sorption processes could offer opportunities in terms of energy consumption and will be pursued as a future research direction.

## Conclusions

In this work, a hollow-fiber membrane module process was successfully simulated for CO_2_ capture, achieving a permeate product that is 125 times more concentrated than the atmospheric air fed to the system. Using the performance results from one of the best experimentally demonstrated membrane materials, a base case simulation was created to process 1 Megaton of air/year. In this case base, the m-DAC process was shown to capture 6.62 tCO_2_/year with a two-staged hollow fiber membrane process. The concentrated product at 5% CO_2_ can be used directly in low-purity CO_2_ processes, such as algal production, catalytic oxidation processes, and enhanced oil recovery. A process operability framework was extensively discussed, which related the input parameters like membrane area, intrinsic properties, and feed flow rate to the overall system properties like product purity, recovery, and energy consumption. Exploring the effect of intrinsic properties’ on the proposed capture process led to results that show that high CO_2_ permeance values are key to achieving high purities and recoveries. Moreover, the selectivity over other mixture components, primarily N_2_ in this particular investigation, affected the energy consumption significantly. The surface areas of the two stages were shown to have opposing effects on purity and energy consumption. Specifically, increasing the area of the first membrane was shown to have an outsized positive impact on all final properties, while a very high surface area in the second membrane module led to greater energy consumption. The process feed loading analysis demonstrated an inversely proportional relationship with the recovery in all cases, increasing the CO_2_ recovery at lower feeds. This reinforces the fact that this process can be better operated under smaller scales (4–40 tons CO_2_/year) and can guide future experimental research and process systems analysis, including optimization efforts and scale-up opportunities. Based on the preliminary CO_2_ emission analysis, operating the proposed m-DAC process consuming power generated exclusively by the current US grid will not be sufficient to enable the NET zero scenario. In contrast, wind and solar power could help to qualify m-DAC as a negative emissions technology, demonstrating that the energy transition to more renewable sources could enable the proposed capture technology. Even though m-DAC is still in its infancy, it could be a promising technology on a small scale. Moreover, future research directions should involve operational parameters such as pressure ratio and alternative designs, including the incorporation of additional modules or a recycle stream, to assess scale-up potential and energy-saving opportunities.

## Supporting Information Available

Additional investigations and data tables for each of the case studies on the effects of the intrinsic membrane properties on (i) isolated first module recovery and energy demands and (ii) isolated second module recovery and energy demands (PDF)
