# Supplementary material for: Process Operability Analysis of Membrane-Based Direct Air Capture for Low-Purity CO2 Production
Source: ACS Eng Au. 2024 Mar 28;4(4):394–404. doi: 10.1021/acsengineeringau.3c00069 (PMC11342364; doi:10.1021/acsengineeringau.3c00069)
Supplement: Supplementary file 1 — eg3c00069_si_001.pdf [file eg3c00069_si_001.pdf]

Process Operability Analysis of Membrane-based Direct Air Capture (m-DAC) for Low Purity CO_2 Production

Vitor Gama, Beatriz Dantas, Oishi Sanyal,* and Fernando V. Lima*

Department of Chemical and Biomedical Engineering, West Virginia University, Morgantown, WV, 26506, USA

E-mail: Oishi.Sanyal@mail.wvu.edu; Fernando.Lima@mail.wvu.edu

Supporting information

Intrinsic properties' studies on isolated modules

In this section, we delve deeper into the role that the membrane properties can play in the context of CO_2 recovery and energy demands. In particular, we have conducted additional research to isolate and understand the complex and subtle effects of these properties on the overall system efficiency. Here, we present detailed plots visualizing the interaction between membrane characteristics, CO_2 recovery and energy consumption. Alongside these plots, we also provide the corresponding data tables. This additional material aims to further explore how specific membrane properties can significantly influence the performance of CO_2 recovery processes, thereby guiding the development of more efficient and sustainable separation technologies for m-DAC.

Effects of the intrinsic properties of the 1st membrane on its immediate permeate

Figures (S1, S2) show capture process would look like if the first stage operated by itself.

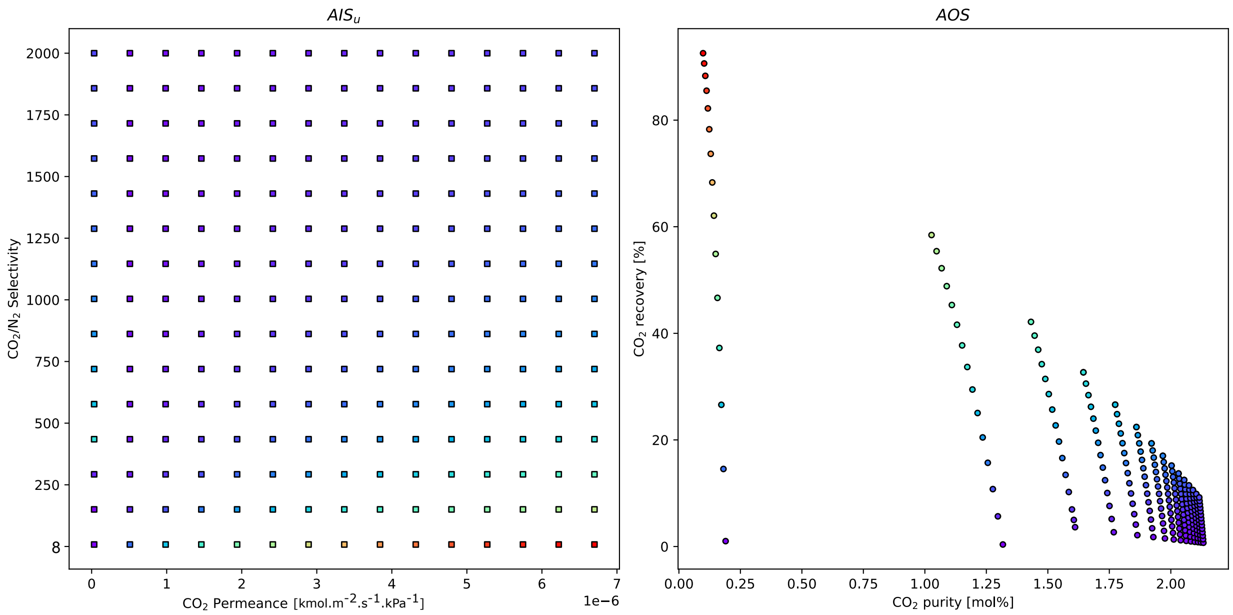

S 1: m-DAC operability study investigating the isolated first membrane module permeance and selectivity influence on CO_2 recovery and purity.

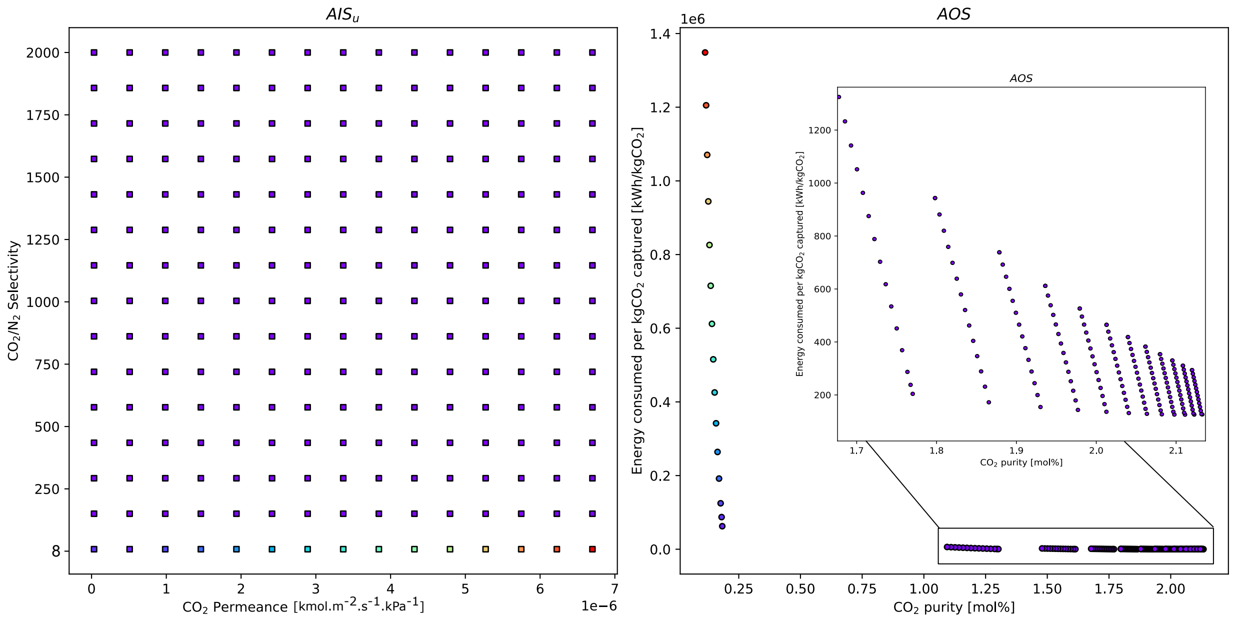

S 2: m-DAC operability study investigating the isolated first membrane module permeance and selectivity influence on CO_2 purity and energy demands.

Effects of the intrisinc properties of the 2nd membrane on its immediate permeate

Figures (S3, S4) demonstrate how the capture process would look like if the first stage operated at its base case conditions (CO_2 permeance = 2,100 GPU (7.0350×10^{-7} kmol.m^{-2}.s^{-1}.kPa^{-1}) and CO_2/N_2 and CO_2/O_2 selectivities of 1,100 and 265), and only the properties of the second module were manipulated.

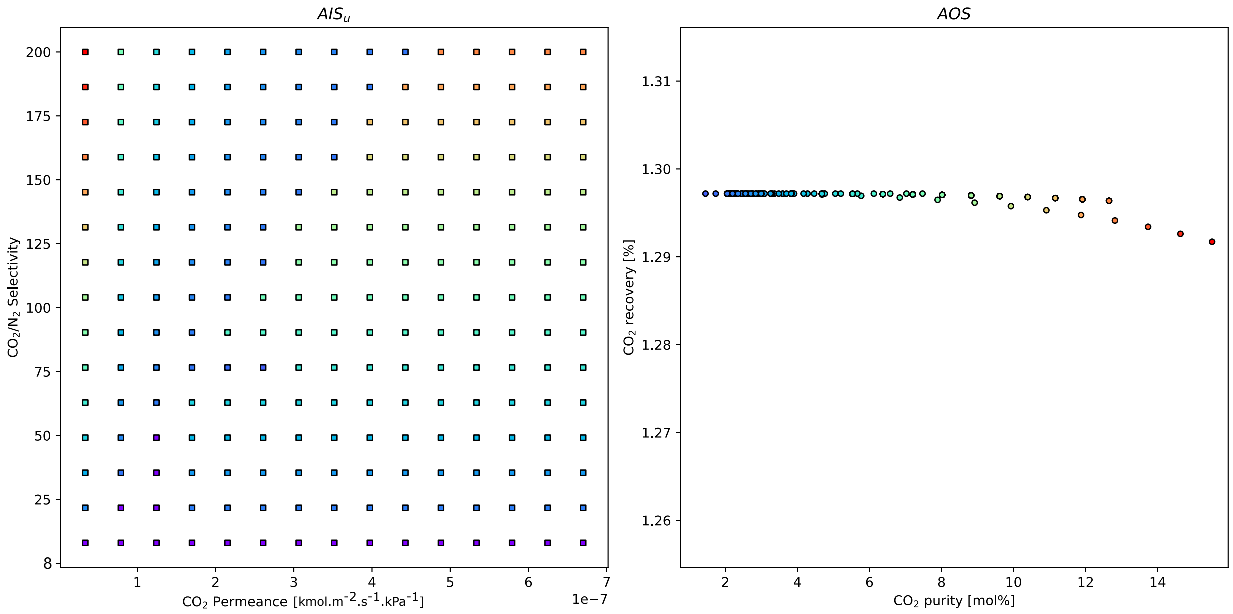

S 3: m-DAC operability study investigating the isolated second membrane module permeance and selectivity influence on CO_2 recovery and purity.

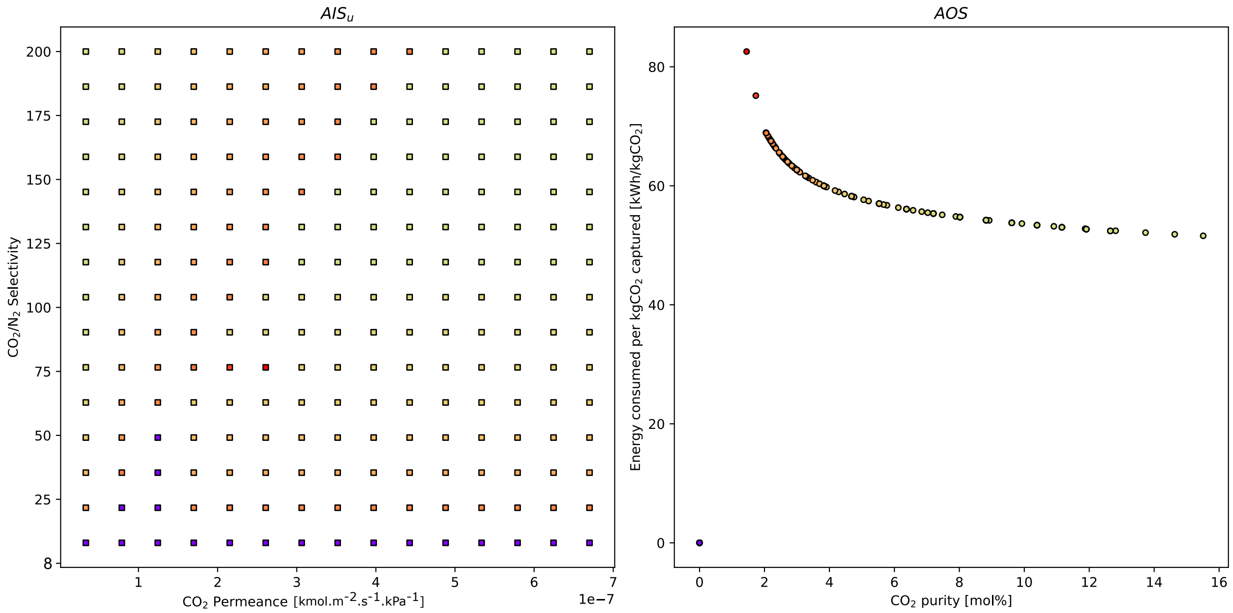

S 4: m-DAC operability study investigating the isolated second membrane module permeance and selectivity influence on CO_2 purity and energy demands.

Operability results

Table 1: Recovery data for effects of the intrinsic properties of the 1st membrane on its immediate permeate

AIS		AOS	
**CO_2 Permeance**	**CO_2/N_2 Selectivity**	**CO_2 purity**	**CO_2 recovery**
3.35E-08	8	0.189975	0.778899
3.35E-08	150.286	1.31749	0.291066
3.35E-08	292.571	1.60997	3.84754
3.35E-08	434.857	1.67772	25.6715
3.35E-08	577.143	1.79814	20.7657
3.35E-08	719.429	1.87865	17.4237
3.35E-08	861.714	1.9362	15.0039
3.35E-08	1004	1.97934	13.1721
3.35E-08	1146.29	2.01288	11.7377
3.35E-08	1288.57	2.03969	10.5844
3.35E-08	1430.86	2.06161	9.63712
3.35E-08	1573.14	2.07986	8.84518
3.35E-08	1715.43	2.0953	8.17335
3.35E-08	1857.71	2.10852	7.59626
3.35E-08	2000	2.11997	7.09521
5.10E-07	8	0.183162	11.3488
5.10E-07	150.286	1.30191	4.37373
5.10E-07	292.571	1.6139	2.79463
5.10E-07	434.857	1.77013	2.06573
5.10E-07	577.143	1.86547	1.64197
5.10E-07	719.429	1.93015	1.36384

5.10E-07	861.714	1.97706	1.1669
5.10E-07	1004	2.01272	1.01998
5.10E-07	1146.29	2.04076	0.906094
5.10E-07	1288.57	2.06342	0.815188
5.10E-07	1430.86	2.08212	0.740924
5.10E-07	1573.14	2.09781	0.679103
5.10E-07	1715.43	2.11118	0.626832
5.10E-07	1857.71	2.1227	0.582053
5.10E-07	2000	2.13274	0.543259
9.86E-07	8	0.176561	21.0179
9.86E-07	150.286	1.28622	8.35373
9.86E-07	292.571	1.60421	5.37159
9.86E-07	434.857	1.76355	3.98006
9.86E-07	577.143	1.86067	3.16739
9.86E-07	719.429	1.92647	2.6327
9.86E-07	861.714	1.97413	2.25355
9.86E-07	1004	2.01031	1.97041
9.86E-07	1146.29	2.03874	1.75078
9.86E-07	1288.57	2.0617	1.57538
9.86E-07	1430.86	2.08062	1.43204
9.86E-07	1573.14	2.0965	1.31268
9.86E-07	1715.43	2.11001	1.21174
9.86E-07	1857.71	2.12166	1.12525
9.86E-07	2000	2.1318	1.05031
1.46E-06	8	0.170172	29.8389
1.46E-06	150.286	1.27044	12.2304

1.46E-06	292.571	1.59439	7.91511
1.46E-06	434.857	1.75688	5.87902
1.46E-06	577.143	1.85581	4.68433
1.46E-06	719.429	1.92274	3.89633
1.46E-06	861.714	1.97116	3.33669
1.46E-06	1004	2.00787	2.91836
1.46E-06	1146.29	2.0367	2.59365
1.46E-06	1288.57	2.05995	2.33419
1.46E-06	1430.86	2.07911	2.12208
1.46E-06	1573.14	2.09517	1.9454
1.46E-06	1715.43	2.10884	1.79595
1.46E-06	1857.71	2.12061	1.66786
1.46E-06	2000	2.13085	1.55687
1.94E-06	8	0.163996	37.8633
1.94E-06	150.286	1.25458	16.003
1.94E-06	292.571	1.58445	10.4246
1.94E-06	434.857	1.75013	7.76232
1.94E-06	577.143	1.85089	6.19262
1.94E-06	719.429	1.91896	5.15461
1.94E-06	861.714	1.96815	4.41629
1.94E-06	1004	2.00542	3.86381
1.94E-06	1146.29	2.03464	3.43467
1.94E-06	1288.57	2.0582	3.0916
1.94E-06	1430.86	2.07759	2.81102
1.94E-06	1573.14	2.09384	2.57724
1.94E-06	1715.43	2.10766	2.37944

1.94E-06	1857.71	2.11955	2.20989
1.94E-06	2000	2.1299	2.06293
2.41E-06	8	0.158033	45.1419
2.41E-06	150.286	1.23865	19.6713
2.41E-06	292.571	1.57439	12.8996
2.41E-06	434.857	1.74328	9.62967
2.41E-06	577.143	1.8459	7.69212
2.41E-06	719.429	1.91514	6.40748
2.41E-06	861.714	1.96512	5.49227
2.41E-06	1004	2.00293	4.80672
2.41E-06	1146.29	2.03256	4.27383
2.41E-06	1288.57	2.05642	3.84759
2.41E-06	1430.86	2.07605	3.49885
2.41E-06	1573.14	2.09249	3.2082
2.41E-06	1715.43	2.10646	2.96221
2.41E-06	1857.71	2.11848	2.75131
2.41E-06	2000	2.12893	2.56849
2.89E-06	8	0.152281	51.724
2.89E-06	150.286	1.22266	23.2348
2.89E-06	292.571	1.56422	15.3396
2.89E-06	434.857	1.73635	11.4808
2.89E-06	577.143	1.84085	9.18267
2.89E-06	719.429	1.91128	7.65483
2.89E-06	861.714	1.96204	6.56458
2.89E-06	1004	2.00042	5.74705
2.89E-06	1146.29	2.03046	5.11109

2.89E-06	1288.57	2.05463	4.60214
2.89E-06	1430.86	2.07451	4.18556
2.89E-06	1573.14	2.09114	3.83825
2.89E-06	1715.43	2.10526	3.54425
2.89E-06	1857.71	2.11741	3.29214
2.89E-06	2000	2.12797	3.07355
3.37E-06	8	0.146742	57.658
3.37E-06	150.286	1.20662	26.6936
3.37E-06	292.571	1.55393	17.7439
3.37E-06	434.857	1.72933	13.3154
3.37E-06	577.143	1.83574	10.6641
3.37E-06	719.429	1.90737	8.89657
3.37E-06	861.714	1.95894	7.63317
3.37E-06	1004	1.99788	6.68477
3.37E-06	1146.29	2.02834	5.94643
3.37E-06	1288.57	2.05283	5.35524
3.37E-06	1430.86	2.07294	4.87113
3.37E-06	1573.14	2.08977	4.46741
3.37E-06	1715.43	2.10405	4.12555
3.37E-06	1857.71	2.11633	3.83235
3.37E-06	2000	2.12699	3.5781
3.84E-06	8	0.141411	62.9903
3.84E-06	150.286	1.19055	30.0476
3.84E-06	292.571	1.54354	20.1121
3.84E-06	434.857	1.72222	15.1332
3.84E-06	577.143	1.83056	12.1363

3.84E-06	719.429	1.90341	10.1326
3.84E-06	861.714	1.9558	8.69797
3.84E-06	1004	1.99531	7.61982
3.84E-06	1146.29	2.0262	6.77983
3.84E-06	1288.57	2.051	6.10685
3.84E-06	1430.86	2.07137	5.55555
3.84E-06	1573.14	2.08839	5.09564
3.84E-06	1715.43	2.10283	4.70611
3.84E-06	1857.71	2.11523	4.37195
3.84E-06	2000	2.12601	4.08213
4.32E-06	8	0.136289	67.7663
4.32E-06	150.286	1.17446	33.2969
4.32E-06	292.571	1.53303	22.4438
4.32E-06	434.857	1.71502	16.9339
4.32E-06	577.143	1.82532	13.599
4.32E-06	719.429	1.8994	11.3629
4.32E-06	861.714	1.95262	9.75893
4.32E-06	1004	1.99272	8.55218
4.32E-06	1146.29	2.02403	7.61124
4.32E-06	1288.57	2.04916	6.85698
4.32E-06	1430.86	2.06978	6.23881
4.32E-06	1573.14	2.087	5.72295
4.32E-06	1715.43	2.1016	5.28592
4.32E-06	1857.71	2.11414	4.91093
4.32E-06	2000	2.12502	4.58564
4.80E-06	8	0.131371	72.0292

4.80E-06	150.286	1.15835	36.442
4.80E-06	292.571	1.52241	24.7384
4.80E-06	434.857	1.70774	18.7173
4.80E-06	577.143	1.82001	15.0522
4.80E-06	719.429	1.89535	12.5872
4.80E-06	861.714	1.94941	10.816
4.80E-06	1004	1.9901	9.48179
4.80E-06	1146.29	2.02185	8.44066
4.80E-06	1288.57	2.0473	7.60558
4.80E-06	1430.86	2.06817	6.92089
4.80E-06	1573.14	2.08559	6.34931
4.80E-06	1715.43	2.10036	5.86496
4.80E-06	1857.71	2.11303	5.44928
4.80E-06	2000	2.12402	5.08863
5.27E-06	8	0.126655	75.8208
5.27E-06	150.286	1.14225	39.4833
5.27E-06	292.571	1.51169	26.9955
5.27E-06	434.857	1.70036	20.483
5.27E-06	577.143	1.81464	16.4956
5.27E-06	719.429	1.89125	13.8056
5.27E-06	861.714	1.94616	11.8691
5.27E-06	1004	1.98745	10.4086
5.27E-06	1146.29	2.01964	9.26805
5.27E-06	1288.57	2.04543	8.35265
5.27E-06	1430.86	2.06655	7.60177
5.27E-06	1573.14	2.08418	6.97473

5.27E-06	1715.43	2.09911	6.44323
5.27E-06	1857.71	2.11191	5.98699
5.27E-06	2000	2.12302	5.59108
5.75E-06	8	0.122138	79.1809
5.75E-06	150.286	1.12616	42.4214
5.75E-06	292.571	1.50086	29.2146
5.75E-06	434.857	1.6929	22.2308
5.75E-06	577.143	1.80921	17.9291
5.75E-06	719.429	1.8871	15.0178
5.75E-06	861.714	1.94288	12.9181
5.75E-06	1004	1.98478	11.3327
5.75E-06	1146.29	2.01741	10.0934
5.75E-06	1288.57	2.04353	9.09817
5.75E-06	1430.86	2.06492	8.28145
5.75E-06	1573.14	2.08275	7.59919
5.75E-06	1715.43	2.09785	7.02073
5.75E-06	1857.71	2.11079	6.52406
5.75E-06	2000	2.12201	6.093
6.22E-06	8	0.117815	82.1471
6.22E-06	150.286	1.1101	45.2573
6.22E-06	292.571	1.48994	31.3955
6.22E-06	434.857	1.68535	23.9604
6.22E-06	577.143	1.8037	19.3525
6.22E-06	719.429	1.8829	16.2239
6.22E-06	861.714	1.93955	13.9631
6.22E-06	1004	1.98208	12.2538

6.22E-06	1146.29	2.01516	10.9166
6.22E-06	1288.57	2.04162	9.84211
6.22E-06	1430.86	2.06327	8.9599
6.22E-06	1573.14	2.08131	8.22268
6.22E-06	1715.43	2.09658	7.59744
6.22E-06	1857.71	2.10966	7.06049
6.22E-06	2000	2.12099	6.59438
6.70E-06	8	0.113683	84.7555
6.70E-06	150.286	1.09408	47.9917
6.70E-06	292.571	1.47892	33.5376
6.70E-06	434.857	1.67772	25.6715
6.70E-06	577.143	1.79814	20.7657
6.70E-06	719.429	1.87865	17.4237
6.70E-06	861.714	1.9362	15.0039
6.70E-06	1004	1.97934	13.1721
6.70E-06	1146.29	2.01288	11.7377
6.70E-06	1288.57	2.03969	10.5844
6.70E-06	1430.86	2.06161	9.63712
6.70E-06	1573.14	2.07986	8.84518
6.70E-06	1715.43	2.0953	8.17335
6.70E-06	1857.71	2.10852	7.59626
6.70E-06	2000	2.11997	7.09521

Table 2: Enery data for effects of the intrinsic properties of the 1st membrane on its immediate permeate

**AIS**		**AOS**	
**CO_2 Permeance**	**CO_2/N_2 Selectivity**	**CO_2 purity**	**Energy consumption ($kWh/kgCO_2$)**
3.35E-08	8	0.18045	87280.4
3.35E-08	150.286	1.29553	668.39
3.35E-08	292.571	1.60997	329.835
3.35E-08	434.857	1.67772	1324.94
3.35E-08	577.143	1.79814	943.268
3.35E-08	719.429	1.87865	738.368
3.35E-08	861.714	1.9362	611.787
3.35E-08	1004	1.97934	526.26
3.35E-08	1146.29	2.01288	464.773
3.35E-08	1288.57	2.03969	418.516
3.35E-08	1430.86	2.06161	382.489
3.35E-08	1573.14	2.07986	353.652
3.35E-08	1715.43	2.0953	330.055
3.35E-08	1857.71	2.10852	310.392
3.35E-08	2000	2.11997	293.754
5.10E-07	8	0.183162	62515.9
5.10E-07	150.286	1.30191	525.813
5.10E-07	292.571	1.6139	273.838
5.10E-07	434.857	1.77013	204.09
5.10E-07	577.143	1.86547	172.195
5.10E-07	719.429	1.93015	154.383

5.10E-07	861.714	1.97706	143.407
5.10E-07	1004	2.01272	136.303
5.10E-07	1146.29	2.04076	131.632
5.10E-07	1288.57	2.06342	128.609
5.10E-07	1430.86	2.08212	126.767
5.10E-07	1573.14	2.09781	125.803
5.10E-07	1715.43	2.11118	125.507
5.10E-07	1857.71	2.1227	125.721
5.10E-07	2000	2.13274	126.317
9.86E-07	8	0.176561	124750
9.86E-07	150.286	1.28622	879.027
9.86E-07	292.571	1.60421	411.147
9.86E-07	434.857	1.76355	286.983
9.86E-07	577.143	1.86067	231.15
9.86E-07	719.429	1.92647	199.578
9.86E-07	861.714	1.97413	179.326
9.86E-07	1004	2.01031	165.295
9.86E-07	1146.29	2.03874	155.071
9.86E-07	1288.57	2.0617	147.361
9.86E-07	1430.86	2.08062	141.405
9.86E-07	1573.14	2.0965	136.728
9.86E-07	1715.43	2.11001	133.015
9.86E-07	1857.71	2.12166	130.05
9.86E-07	2000	2.1318	127.679
1.46E-06	8	0.170172	191849
1.46E-06	150.286	1.27044	1243.02

1.46E-06	292.571	1.59439	549.34
1.46E-06	434.857	1.75688	368.848
1.46E-06	577.143	1.85581	288.94
1.46E-06	719.429	1.92274	244.294
1.46E-06	861.714	1.97116	215.838
1.46E-06	1004	2.00787	196.111
1.46E-06	1146.29	2.0367	181.631
1.46E-06	1288.57	2.05995	170.56
1.46E-06	1430.86	2.07911	161.84
1.46E-06	1573.14	2.09517	154.816
1.46E-06	1715.43	2.10884	149.061
1.46E-06	1857.71	2.12061	144.281
1.46E-06	2000	2.13085	140.27
1.94E-06	8	0.163996	264135
1.94E-06	150.286	1.25458	1618.55
1.94E-06	292.571	1.58445	689.641
1.94E-06	434.857	1.75013	451.089
1.94E-06	577.143	1.85089	346.432
1.94E-06	719.429	1.91896	288.42
1.94E-06	861.714	1.96815	251.703
1.94E-06	1004	2.00542	226.387
1.94E-06	1146.29	2.03464	207.865
1.94E-06	1288.57	2.0582	193.712
1.94E-06	1430.86	2.07759	182.54
1.94E-06	1573.14	2.09384	173.499
1.94E-06	1715.43	2.10766	166.036

1.94E-06	1857.71	2.11955	159.78
1.94E-06	2000	2.1299	154.469
2.41E-06	8	0.158033	341941
2.41E-06	150.286	1.23865	2006.14
2.41E-06	292.571	1.57439	832.293
2.41E-06	434.857	1.74328	534.085
2.41E-06	577.143	1.8459	404.109
2.41E-06	719.429	1.91514	332.437
2.41E-06	861.714	1.96512	287.29
2.41E-06	1004	2.00293	256.3
2.41E-06	1146.29	2.03256	233.713
2.41E-06	1288.57	2.05642	216.507
2.41E-06	1430.86	2.07605	202.952
2.41E-06	1573.14	2.09249	191.988
2.41E-06	1715.43	2.10646	182.933
2.41E-06	1857.71	2.11848	175.326
2.41E-06	2000	2.12893	168.848
2.89E-06	8	0.152281	425609
2.89E-06	150.286	1.22266	2406.3
2.89E-06	292.571	1.56422	977.408
2.89E-06	434.857	1.73635	617.953
2.89E-06	577.143	1.84085	462.138
2.89E-06	719.429	1.91128	376.557
2.89E-06	861.714	1.96204	322.829
2.89E-06	1004	2.00042	286.066
2.89E-06	1146.29	2.03046	259.352

2.89E-06	1288.57	2.05463	239.058
2.89E-06	1430.86	2.07451	223.109
2.89E-06	1573.14	2.09114	210.234
2.89E-06	1715.43	2.10526	199.614
2.89E-06	1857.71	2.11741	190.698
2.89E-06	2000	2.12797	183.102
3.37E-06	8	0.146742	515488
3.37E-06	150.286	1.20662	2819.58
3.37E-06	292.571	1.55393	1125.08
3.37E-06	434.857	1.72933	702.746
3.37E-06	577.143	1.83574	520.584
3.37E-06	719.429	1.90737	420.866
3.37E-06	861.714	1.95894	358.429
3.37E-06	1004	1.99788	315.807
3.37E-06	1146.29	2.02834	284.904
3.37E-06	1288.57	2.05283	261.481
3.37E-06	1430.86	2.07294	243.109
3.37E-06	1573.14	2.08977	228.307
3.37E-06	1715.43	2.10405	216.116
3.37E-06	1857.71	2.11633	205.895
3.37E-06	2000	2.12699	197.196
3.84E-06	8	0.141411	611933
3.84E-06	150.286	1.19055	3246.54
3.84E-06	292.571	1.54354	1275.39
3.84E-06	434.857	1.72222	788.5
3.84E-06	577.143	1.83056	579.481

3.84E-06	719.429	1.90341	465.408
3.84E-06	861.714	1.9558	394.142
3.84E-06	1004	1.99531	345.587
3.84E-06	1146.29	2.0262	310.443
3.84E-06	1288.57	2.051	283.849
3.84E-06	1430.86	2.07137	263.025
3.84E-06	1573.14	2.08839	246.272
3.84E-06	1715.43	2.10283	232.496
3.84E-06	1857.71	2.11523	220.962
3.84E-06	2000	2.12601	211.156
4.32E-06	8	0.136289	715302
4.32E-06	150.286	1.17446	3687.76
4.32E-06	292.571	1.53303	1428.44
4.32E-06	434.857	1.71502	875.246
4.32E-06	577.143	1.82532	638.85
4.32E-06	719.429	1.8994	510.203
4.32E-06	861.714	1.95262	429.997
4.32E-06	1004	1.99272	375.439
4.32E-06	1146.29	2.02403	336.007
4.32E-06	1288.57	2.04916	306.207
4.32E-06	1430.86	2.06978	282.903
4.32E-06	1573.14	2.087	264.179
4.32E-06	1715.43	2.1016	248.802
4.32E-06	1857.71	2.11414	235.941
4.32E-06	2000	2.12502	225.02
4.80E-06	8	0.131371	825957

4.80E-06	150.286	1.15835	4143.82
4.80E-06	292.571	1.52241	1584.33
4.80E-06	434.857	1.70774	963.015
4.80E-06	577.143	1.82001	698.705
4.80E-06	719.429	1.89535	555.265
4.80E-06	861.714	1.94941	466.009
4.80E-06	1004	1.9901	405.383
4.80E-06	1146.29	2.02185	361.619
4.80E-06	1288.57	2.0473	328.583
4.80E-06	1430.86	2.06817	302.775
4.80E-06	1573.14	2.08559	282.061
4.80E-06	1715.43	2.10036	265.065
4.80E-06	1857.71	2.11303	250.865
4.80E-06	2000	2.12402	238.819
5.27E-06	8	0.126655	944255
5.27E-06	150.286	1.14225	4615.34
5.27E-06	292.571	1.51169	1743.15
5.27E-06	434.857	1.70036	1051.84
5.27E-06	577.143	1.81464	759.061
5.27E-06	719.429	1.89125	600.606
5.27E-06	861.714	1.94616	502.187
5.27E-06	1004	1.98745	435.43
5.27E-06	1146.29	2.01964	387.295
5.27E-06	1288.57	2.04543	350.993
5.27E-06	1430.86	2.06655	322.659
5.27E-06	1573.14	2.08418	299.937

5.27E-06	1715.43	2.09911	281.309
5.27E-06	1857.71	2.11191	265.759
5.27E-06	2000	2.12302	252.577
5.75E-06	8	0.122138	1.07E+06
5.75E-06	150.286	1.12616	5102.92
5.75E-06	292.571	1.50086	1905
5.75E-06	434.857	1.6929	1141.74
5.75E-06	577.143	1.80921	819.931
5.75E-06	719.429	1.8871	646.231
5.75E-06	861.714	1.94288	538.54
5.75E-06	1004	1.98478	465.589
5.75E-06	1146.29	2.01741	413.042
5.75E-06	1288.57	2.04353	373.448
5.75E-06	1430.86	2.06492	342.568
5.75E-06	1573.14	2.08275	317.822
5.75E-06	1715.43	2.09785	297.549
5.75E-06	1857.71	2.11079	280.637
5.75E-06	2000	2.12201	266.312
6.22E-06	8	0.117815	1.21E+06
6.22E-06	150.286	1.1101	5607.18
6.22E-06	292.571	1.48994	2069.99
6.22E-06	434.857	1.68535	1232.77
6.22E-06	577.143	1.8037	881.329
6.22E-06	719.429	1.8829	692.15
6.22E-06	861.714	1.93955	575.071
6.22E-06	1004	1.98208	495.864

6.22E-06	1146.29	2.01516	438.867
6.22E-06	1288.57	2.04162	395.954
6.22E-06	1430.86	2.06327	362.509
6.22E-06	1573.14	2.08131	335.725
6.22E-06	1715.43	2.09658	313.796
6.22E-06	1857.71	2.10966	295.512
6.22E-06	2000	2.12099	280.034
6.70E-06	8	0.113683	1.35E+06
6.70E-06	150.286	1.09408	6128.77
6.70E-06	292.571	1.47892	2238.22
6.70E-06	434.857	1.67772	1324.94
6.70E-06	577.143	1.79814	943.268
6.70E-06	719.429	1.87865	738.368
6.70E-06	861.714	1.9362	611.787
6.70E-06	1004	1.97934	526.26
6.70E-06	1146.29	2.01288	464.773
6.70E-06	1288.57	2.03969	418.516
6.70E-06	1430.86	2.06161	382.489
6.70E-06	1573.14	2.07986	353.652
6.70E-06	1715.43	2.0953	330.055
6.70E-06	1857.71	2.10852	310.392
6.70E-06	2000	2.11997	293.754

Table 3: Recovery data for effects of the intrinsic properties of the 2nd membrane on its immediate permeate

**AIS**		**AOS**	
**CO_2 Permeance**	**CO_2/N_2 Selectivity**	**CO_2 purity**	**CO_2 recovery**
3.35E-08	8	0.189975	0.778899
3.35E-08	8	0	0
3.35E-08	21.7143	2.55056	1.29719
3.35E-08	35.4286	3.59694	1.29717
3.35E-08	49.1429	4.68515	1.29709
3.35E-08	62.8571	5.77111	1.29694
3.35E-08	76.5714	6.84135	1.29674
3.35E-08	90.2857	7.89147	1.29648
3.35E-08	104	8.91991	1.29616
3.35E-08	117.714	9.92621	1.29576
3.35E-08	131.429	10.9104	1.2953
3.35E-08	145.143	11.8726	1.29475
3.35E-08	158.857	12.8132	1.29413
3.35E-08	172.571	13.7326	1.29342
3.35E-08	186.286	14.6312	1.29261
3.35E-08	200	15.5094	1.29171
7.90E-08	8	0	0
7.90E-08	21.7143	0	0
7.90E-08	35.4286	2.04543	1.29719
7.90E-08	49.1429	2.48527	1.29719
7.90E-08	62.8571	2.91623	1.29719
7.90E-08	76.5714	3.36307	1.29719

7.90E-08	90.2857	3.81999	1.29719
7.90E-08	104	4.28134	1.29719
7.90E-08	117.714	4.74379	1.29719
7.90E-08	131.429	5.20537	1.29719
7.90E-08	145.143	5.66496	1.29719
7.90E-08	158.857	6.12185	1.29719
7.90E-08	172.571	6.5756	1.29719
7.90E-08	186.286	7.02594	1.29719
7.90E-08	200	7.47268	1.29719
1.24E-07	8	0	0
1.24E-07	21.7143	0	0
1.24E-07	35.4286	0	0
1.24E-07	49.1429	0	0
1.24E-07	62.8571	2.1948	1.29719
1.24E-07	76.5714	2.46774	1.29719
1.24E-07	90.2857	2.7389	1.29719
1.24E-07	104	3.01662	1.29719
1.24E-07	117.714	3.3006	1.29719
1.24E-07	131.429	3.58899	1.29719
1.24E-07	145.143	3.88012	1.29719
1.24E-07	158.857	4.17277	1.29719
1.24E-07	172.571	4.46608	1.29719
1.24E-07	186.286	4.75944	1.29719
1.24E-07	200	5.05243	1.29719
1.70E-07	8	0	0
1.70E-07	21.7143	2.19943	1.29719

1.70E-07	35.4286	2.9946	1.29719
1.70E-07	49.1429	3.82951	1.29719
1.70E-07	62.8571	4.68072	1.29718
1.70E-07	76.5714	2.05042	1.29719
1.70E-07	90.2857	2.26097	1.29719
1.70E-07	104	2.45959	1.29719
1.70E-07	117.714	2.65752	1.29719
1.70E-07	131.429	2.85865	1.29719
1.70E-07	145.143	3.06354	1.29719
1.70E-07	158.857	3.27165	1.29719
1.70E-07	172.571	3.48221	1.29719
1.70E-07	186.286	3.69454	1.29719
1.70E-07	200	3.90807	1.29719
2.15E-07	8	0	0
2.15E-07	21.7143	2.19943	1.29719
2.15E-07	35.4286	2.9946	1.29719
2.15E-07	49.1429	3.82951	1.29719
2.15E-07	62.8571	4.68072	1.29718
2.15E-07	76.5714	1.73337	1.29719
2.15E-07	90.2857	6.37137	1.29713
2.15E-07	104	2.13686	1.29719
2.15E-07	117.714	2.29861	1.29719
2.15E-07	131.429	2.45489	1.29719
2.15E-07	145.143	2.61078	1.29719
2.15E-07	158.857	2.76843	1.29719
2.15E-07	172.571	2.92844	1.29719

2.15E-07	186.286	3.09072	1.29719
2.15E-07	200	3.25495	1.29719
2.61E-07	8	0	0
2.61E-07	21.7143	2.19943	1.29719
2.61E-07	35.4286	2.9946	1.29719
2.61E-07	49.1429	3.82951	1.29719
2.61E-07	62.8571	4.68072	1.29718
2.61E-07	76.5714	5.53027	1.29716
2.61E-07	90.2857	6.37137	1.29713
2.61E-07	104	7.20124	1.2971
2.61E-07	117.714	2.05193	1.29719
2.61E-07	131.429	2.19116	1.29719
2.61E-07	145.143	2.32297	1.29719
2.61E-07	158.857	2.45183	1.29719
2.61E-07	172.571	2.58042	1.29719
2.61E-07	186.286	2.71008	1.29719
2.61E-07	200	2.84129	1.29719
3.06E-07	8	0	0
3.06E-07	21.7143	2.19943	1.29719
3.06E-07	35.4286	2.9946	1.29719
3.06E-07	49.1429	3.82951	1.29719
3.06E-07	62.8571	4.68072	1.29718
3.06E-07	76.5714	5.53027	1.29716
3.06E-07	90.2857	6.37137	1.29713
3.06E-07	104	7.20124	1.2971
3.06E-07	117.714	8.01865	1.29705

3.06E-07	131.429	8.82309	1.29698
3.06E-07	145.143	2.11287	1.29719
3.06E-07	158.857	2.22861	1.29719
3.06E-07	172.571	2.34004	1.29719
3.06E-07	186.286	2.44967	1.29719
3.06E-07	200	2.55912	1.29719
3.52E-07	8	0	0
3.52E-07	21.7143	2.19943	1.29719
3.52E-07	35.4286	2.9946	1.29719
3.52E-07	49.1429	3.82951	1.29719
3.52E-07	62.8571	4.68072	1.29718
3.52E-07	76.5714	5.53027	1.29716
3.52E-07	90.2857	6.37137	1.29713
3.52E-07	104	7.20124	1.2971
3.52E-07	117.714	8.01865	1.29705
3.52E-07	131.429	8.82309	1.29698
3.52E-07	145.143	9.61439	1.2969
3.52E-07	158.857	2.05266	1.29719
3.52E-07	172.571	2.15677	1.29719
3.52E-07	186.286	2.25608	1.29719
3.52E-07	200	2.35266	1.29719
3.97E-07	8	0	0
3.97E-07	21.7143	2.19943	1.29719
3.97E-07	35.4286	2.9946	1.29719
3.97E-07	49.1429	3.82951	1.29719
3.97E-07	62.8571	4.68072	1.29718

3.97E-07	76.5714	5.53027	1.29716
3.97E-07	90.2857	6.37137	1.29713
3.97E-07	104	7.20124	1.2971
3.97E-07	117.714	8.01865	1.29705
3.97E-07	131.429	8.82309	1.29698
3.97E-07	145.143	9.61439	1.2969
3.97E-07	158.857	10.3925	1.2968
3.97E-07	172.571	11.1576	1.29668
3.97E-07	186.286	2.09973	1.29719
3.97E-07	200	2.19002	1.29719
4.43E-07	8	0	0
4.43E-07	21.7143	2.19943	1.29719
4.43E-07	35.4286	2.9946	1.29719
4.43E-07	49.1429	3.82951	1.29719
4.43E-07	62.8571	4.68072	1.29718
4.43E-07	76.5714	5.53027	1.29716
4.43E-07	90.2857	6.37137	1.29713
4.43E-07	104	7.20124	1.2971
4.43E-07	117.714	8.01865	1.29705
4.43E-07	131.429	8.82309	1.29698
4.43E-07	145.143	9.61439	1.2969
4.43E-07	158.857	10.3925	1.2968
4.43E-07	172.571	11.1576	1.29668
4.43E-07	186.286	11.9098	1.29654
4.43E-07	200	2.05309	1.29719
4.88E-07	8	0	0

4.88E-07	21.7143	2.19943	1.29719
4.88E-07	35.4286	2.9946	1.29719
4.88E-07	49.1429	3.82951	1.29719
4.88E-07	62.8571	4.68072	1.29718
4.88E-07	76.5714	5.53027	1.29716
4.88E-07	90.2857	6.37137	1.29713
4.88E-07	104	7.20124	1.2971
4.88E-07	117.714	8.01865	1.29705
4.88E-07	131.429	8.82309	1.29698
4.88E-07	145.143	9.61439	1.2969
4.88E-07	158.857	10.3925	1.2968
4.88E-07	172.571	11.1576	1.29668
4.88E-07	186.286	11.9098	1.29654
4.88E-07	200	12.6493	1.29637
5.34E-07	8	0	0
5.34E-07	21.7143	2.19943	1.29719
5.34E-07	35.4286	2.9946	1.29719
5.34E-07	49.1429	3.82951	1.29719
5.34E-07	62.8571	4.68072	1.29718
5.34E-07	76.5714	5.53027	1.29716
5.34E-07	90.2857	6.37137	1.29713
5.34E-07	104	7.20124	1.2971
5.34E-07	117.714	8.01865	1.29705
5.34E-07	131.429	8.82309	1.29698
5.34E-07	145.143	9.61439	1.2969
5.34E-07	158.857	10.3925	1.2968

5.34E-07	172.571	11.1576	1.29668
5.34E-07	186.286	11.9098	1.29654
5.34E-07	200	12.6493	1.29637
5.79E-07	8	0	0
5.79E-07	21.7143	2.19943	1.29719
5.79E-07	35.4286	2.9946	1.29719
5.79E-07	49.1429	3.82951	1.29719
5.79E-07	62.8571	4.68072	1.29718
5.79E-07	76.5714	5.53027	1.29716
5.79E-07	90.2857	6.37137	1.29713
5.79E-07	104	7.20124	1.2971
5.79E-07	117.714	8.01865	1.29705
5.79E-07	131.429	8.82309	1.29698
5.79E-07	145.143	9.61439	1.2969
5.79E-07	158.857	10.3925	1.2968
5.79E-07	172.571	11.1576	1.29668
5.79E-07	186.286	11.9098	1.29654
5.79E-07	200	12.6493	1.29637
6.25E-07	8	0	0
6.25E-07	21.7143	2.19943	1.29719
6.25E-07	35.4286	2.9946	1.29719
6.25E-07	49.1429	3.82951	1.29719
6.25E-07	62.8571	4.68072	1.29718
6.25E-07	76.5714	5.53027	1.29716
6.25E-07	90.2857	6.37137	1.29713
6.25E-07	104	7.20124	1.2971

6.25E-07	117.714	8.01865	1.29705
6.25E-07	131.429	8.82309	1.29698
6.25E-07	145.143	9.61439	1.2969
6.25E-07	158.857	10.3925	1.2968
6.25E-07	172.571	11.1576	1.29668
6.25E-07	186.286	11.9098	1.29654
6.25E-07	200	12.6493	1.29637
6.70E-07	8	0	0
6.70E-07	21.7143	2.19943	1.29719
6.70E-07	35.4286	2.9946	1.29719
6.70E-07	49.1429	3.82951	1.29719
6.70E-07	62.8571	4.68072	1.29718
6.70E-07	76.5714	5.53027	1.29716
6.70E-07	90.2857	6.37137	1.29713
6.70E-07	104	7.20124	1.2971
6.70E-07	117.714	8.01865	1.29705
6.70E-07	131.429	8.82309	1.29698
6.70E-07	145.143	9.61439	1.2969
6.70E-07	158.857	10.3925	1.2968
6.70E-07	172.571	11.1576	1.29668
6.70E-07	186.286	11.9098	1.29654
6.70E-07	200	12.6493	1.29637

Table 4: Energy data for effects of the intrinsic properties of the 2nd membrane on its immediate permeate

**AIS**		**AOS**	
**CO_2 Permeance**	**CO_2/N_2 Selectivity**	**CO_2 purity**	**Energy consumption (kWh/kgCO_2)**
3.35E-08	8	0	0
3.35E-08	21.7143	2.55056	64.9486
3.35E-08	35.4286	3.59694	60.6067
3.35E-08	49.1429	4.68515	58.2417
3.35E-08	62.8571	5.77111	56.731
3.35E-08	76.5714	6.84135	55.6602
3.35E-08	90.2857	7.89147	54.8455
3.35E-08	104	8.91991	54.1941
3.35E-08	117.714	9.92621	53.6541
3.35E-08	131.429	10.9104	53.1945
3.35E-08	145.143	11.8726	52.7958
3.35E-08	158.857	12.8132	52.4446
3.35E-08	172.571	13.7326	52.1321
3.35E-08	186.286	14.6312	51.8515
3.35E-08	200	15.5094	51.5982
7.90E-08	8	0	0
7.90E-08	21.7143	0	0
7.90E-08	35.4286	2.04543	68.9403
7.90E-08	49.1429	2.48527	65.3647
7.90E-08	62.8571	2.91623	63.0239
7.90E-08	76.5714	3.36307	61.3167

7.90E-08	90.2857	3.81999	60.0115
7.90E-08	104	4.28134	58.9792
7.90E-08	117.714	4.74379	58.1397
7.90E-08	131.429	5.20537	57.4405
7.90E-08	145.143	5.66496	56.8466
7.90E-08	158.857	6.12185	56.3333
7.90E-08	172.571	6.5756	55.8833
7.90E-08	186.286	7.02594	55.4838
7.90E-08	200	7.47268	55.1253
1.24E-07	8	0	0
1.24E-07	21.7143	0	0
1.24E-07	35.4286	0	0
1.24E-07	49.1429	0	0
1.24E-07	62.8571	2.1948	67.5675
1.24E-07	76.5714	2.46774	65.481
1.24E-07	90.2857	2.7389	63.8803
1.24E-07	104	3.01662	62.5907
1.24E-07	117.714	3.3006	61.5247
1.24E-07	131.429	3.58899	60.6282
1.24E-07	145.143	3.88012	59.8631
1.24E-07	158.857	4.17277	59.2019
1.24E-07	172.571	4.46608	58.6241
1.24E-07	186.286	4.75944	58.1139
1.24E-07	200	5.05243	57.6593
1.70E-07	8	0	0
1.70E-07	21.7143	2.19943	67.5277

1.70E-07	35.4286	2.9946	62.6831
1.70E-07	49.1429	3.82951	59.9879
1.70E-07	62.8571	4.68072	58.2455
1.70E-07	76.5714	2.05042	68.8919
1.70E-07	90.2857	2.26097	67.0119
1.70E-07	104	2.45959	65.5357
1.70E-07	117.714	2.65752	64.3193
1.70E-07	131.429	2.85865	63.2882
1.70E-07	145.143	3.06354	62.3993
1.70E-07	158.857	3.27165	61.6241
1.70E-07	172.571	3.48221	60.9418
1.70E-07	186.286	3.69454	60.3365
1.70E-07	200	3.90807	59.7957
2.15E-07	8	0	0
2.15E-07	21.7143	2.19943	67.5277
2.15E-07	35.4286	2.9946	62.6831
2.15E-07	49.1429	3.82951	59.9879
2.15E-07	62.8571	4.68072	58.2455
2.15E-07	76.5714	1.73337	75.1522
2.15E-07	90.2857	6.37137	56.0815
2.15E-07	104	2.13686	68.0809
2.15E-07	117.714	2.29861	66.7103
2.15E-07	131.429	2.45489	65.5674
2.15E-07	145.143	2.61078	64.5865
2.15E-07	158.857	2.76843	63.7286
2.15E-07	172.571	2.92844	62.9693

2.15E-07	186.286	3.09072	62.2914
2.15E-07	200	3.25495	61.6822
2.61E-07	8	0	0
2.61E-07	21.7143	2.19943	67.5277
2.61E-07	35.4286	2.9946	62.6831
2.61E-07	49.1429	3.82951	59.9879
2.61E-07	62.8571	4.68072	58.2455
2.61E-07	76.5714	1.44911	82.5646
2.61E-07	90.2857	6.37137	56.0815
2.61E-07	104	7.20124	55.3427
2.61E-07	117.714	2.05193	68.8774
2.61E-07	131.429	2.19116	67.599
2.61E-07	145.143	2.32297	66.5207
2.61E-07	158.857	2.45183	65.5882
2.61E-07	172.571	2.58042	64.7662
2.61E-07	186.286	2.71008	64.0321
2.61E-07	200	2.84129	63.3703
3.06E-07	8	0	0
3.06E-07	21.7143	2.19943	67.5277
3.06E-07	35.4286	2.9946	62.6831
3.06E-07	49.1429	3.82951	59.9879
3.06E-07	62.8571	4.68072	58.2455
3.06E-07	76.5714	5.53027	57.0128
3.06E-07	90.2857	6.37137	56.0815
3.06E-07	104	7.20124	55.3427
3.06E-07	117.714	8.01865	54.7345

3.06E-07	131.429	8.82309	54.2195
3.06E-07	145.143	2.11287	68.3008
3.06E-07	158.857	2.22861	67.2794
3.06E-07	172.571	2.34004	66.3905
3.06E-07	186.286	2.44967	65.6028
3.06E-07	200	2.55912	64.8955
3.52E-07	8	0	0
3.52E-07	21.7143	2.19943	67.5277
3.52E-07	35.4286	2.9946	62.6831
3.52E-07	49.1429	3.82951	59.9879
3.52E-07	62.8571	4.68072	58.2455
3.52E-07	76.5714	5.53027	57.0128
3.52E-07	90.2857	6.37137	56.0815
3.52E-07	104	7.20124	55.3427
3.52E-07	117.714	8.01865	54.7345
3.52E-07	131.429	8.82309	54.2195
3.52E-07	145.143	9.61439	53.7734
3.52E-07	158.857	2.05266	68.8703
3.52E-07	172.571	2.15677	67.9017
3.52E-07	186.286	2.25608	67.0517
3.52E-07	200	2.35266	66.2955
3.97E-07	8	0	0
3.97E-07	21.7143	2.19943	67.5277
3.97E-07	35.4286	2.9946	62.6831
3.97E-07	49.1429	3.82951	59.9879
3.97E-07	62.8571	4.68072	58.2455

3.97E-07	76.5714	5.53027	57.0128
3.97E-07	90.2857	6.37137	56.0815
3.97E-07	104	7.20124	55.3427
3.97E-07	117.714	8.01865	54.7345
3.97E-07	131.429	8.82309	54.2195
3.97E-07	145.143	9.61439	53.7734
3.97E-07	158.857	10.3925	53.3802
3.97E-07	172.571	11.1576	53.0288
3.97E-07	186.286	2.09973	68.423
3.97E-07	200	2.19002	67.6089
4.43E-07	8	0	0
4.43E-07	21.7143	2.19943	67.5277
4.43E-07	35.4286	2.9946	62.6831
4.43E-07	49.1429	3.82951	59.9879
4.43E-07	62.8571	4.68072	58.2455
4.43E-07	76.5714	5.53027	57.0128
4.43E-07	90.2857	6.37137	56.0815
4.43E-07	104	7.20124	55.3427
4.43E-07	117.714	8.01865	54.7345
4.43E-07	131.429	8.82309	54.2195
4.43E-07	145.143	9.61439	53.7734
4.43E-07	158.857	10.3925	53.3802
4.43E-07	172.571	11.1576	53.0288
4.43E-07	186.286	11.9098	52.711
4.43E-07	200	2.05309	68.8662
4.88E-07	8	0	0

4.88E-07	21.7143	2.19943	67.5277
4.88E-07	35.4286	2.9946	62.6831
4.88E-07	49.1429	3.82951	59.9879
4.88E-07	62.8571	4.68072	58.2455
4.88E-07	76.5714	5.53027	57.0128
4.88E-07	90.2857	6.37137	56.0815
4.88E-07	104	7.20124	55.3427
4.88E-07	117.714	8.01865	54.7345
4.88E-07	131.429	8.82309	54.2195
4.88E-07	145.143	9.61439	53.7734
4.88E-07	158.857	10.3925	53.3802
4.88E-07	172.571	11.1576	53.0288
4.88E-07	186.286	11.9098	52.711
4.88E-07	200	12.6493	52.4211
5.34E-07	8	0	0
5.34E-07	21.7143	2.19943	67.5277
5.34E-07	35.4286	2.9946	62.6831
5.34E-07	49.1429	3.82951	59.9879
5.34E-07	62.8571	4.68072	58.2455
5.34E-07	76.5714	5.53027	57.0128
5.34E-07	90.2857	6.37137	56.0815
5.34E-07	104	7.20124	55.3427
5.34E-07	117.714	8.01865	54.7345
5.34E-07	131.429	8.82309	54.2195
5.34E-07	145.143	9.61439	53.7734
5.34E-07	158.857	10.3925	53.3802

5.34E-07	172.571	11.1576	53.0288
5.34E-07	186.286	11.9098	52.711
5.34E-07	200	12.6493	52.4211
5.79E-07	8	0	0
5.79E-07	21.7143	2.19943	67.5277
5.79E-07	35.4286	2.9946	62.6831
5.79E-07	49.1429	3.82951	59.9879
5.79E-07	62.8571	4.68072	58.2455
5.79E-07	76.5714	5.53027	57.0128
5.79E-07	90.2857	6.37137	56.0815
5.79E-07	104	7.20124	55.3427
5.79E-07	117.714	8.01865	54.7345
5.79E-07	131.429	8.82309	54.2195
5.79E-07	145.143	9.61439	53.7734
5.79E-07	158.857	10.3925	53.3802
5.79E-07	172.571	11.1576	53.0288
5.79E-07	186.286	11.9098	52.711
5.79E-07	200	12.6493	52.4211
6.25E-07	8	0	0
6.25E-07	21.7143	2.19943	67.5277
6.25E-07	35.4286	2.9946	62.6831
6.25E-07	49.1429	3.82951	59.9879
6.25E-07	62.8571	4.68072	58.2455
6.25E-07	76.5714	5.53027	57.0128
6.25E-07	90.2857	6.37137	56.0815
6.25E-07	104	7.20124	55.3427

6.25E-07	117.714	8.01865	54.7345
6.25E-07	131.429	8.82309	54.2195
6.25E-07	145.143	9.61439	53.7734
6.25E-07	158.857	10.3925	53.3802
6.25E-07	172.571	11.1576	53.0288
6.25E-07	186.286	11.9098	52.711
6.25E-07	200	12.6493	52.4211
6.70E-07	8	0	0
6.70E-07	21.7143	2.19943	67.5277
6.70E-07	35.4286	2.9946	62.6831
6.70E-07	49.1429	3.82951	59.9879
6.70E-07	62.8571	4.68072	58.2455
6.70E-07	76.5714	5.53027	57.0128
6.70E-07	90.2857	6.37137	56.0815
6.70E-07	104	7.20124	55.3427
6.70E-07	117.714	8.01865	54.7345
6.70E-07	131.429	8.82309	54.2195
6.70E-07	145.143	9.61439	53.7734
6.70E-07	158.857	10.3925	53.3802
6.70E-07	172.571	11.1576	53.0288
6.70E-07	186.286	11.9098	52.711
6.70E-07	200	12.6493	52.4211
